# Isoxazolyl-Derived 1,4-Dihydroazolo[5,1-*c*][1,2,4]Triazines: Synthesis and Photochemical Properties

**DOI:** 10.3390/molecules28073192

**Published:** 2023-04-03

**Authors:** Elena V. Sadchikova, Nikita E. Safronov, Nikolai A. Beliaev, Valentine G. Nenajdenko, Nataliya P. Belskaya

**Affiliations:** 1 Department of Technology for Organic Synthesis, Ural Federal University, 620002 Ekaterinburg, Russia; 2 Department of Organic Chemistry, Moscow State University, 119992 Moscow, Russia

**Keywords:** dihydroazolo[5,1-*c*][1,2,4]triazines, diazopyrazole, diazoimidazole, enamine, isoxazole, fluorescence, solvatochromism, AIE effect

## Abstract

New fluorescent dyes containing an assembled 1,4-dihydroazolo[5,1-*c*][1,2,4]triazine (DAT) core and an isoxazole ring were synthesized through a reaction between diazopyrazole or diazoimidazoles and isoxazolyl-derived enamines in mild conditions. The photophysical characteristics (maxima absorption and emission, Stokes shifts, fluorescent quantum yields, and fluorescence lifetimes) of the new fluorophores were obtained. The prepared DATs demonstrated emission maxima ranging within 433–487 nm, quantum yields within 6.1–33.3%, and a large Stokes shift. The photophysical characteristics of representative DAT examples were studied in ten different solvents. Specific (hydrogen bonds) and non-specific (dipole–dipole) intermolecular and intramolecular interactions were analyzed using XRD data and spectral experiments. Solvatochromism was analyzed using Lippert–Mataga and Dimroth–Reichardt plots, revealing the relationship between the DAT structure and the nature of solute–solvent interactions. The significant advantages of DATs are the fluorescence of their powders (QY up to 98.7%). DAT-NMe_2_ **10** expressed bright aggregation-induced emission (AIE) behavior in DMSO and THF as the water content increased. The numerous possible variations of the structures of the heterocycles included in the DATs, as well as substituents, create excellent prospects for adjusting their photophysical and physicochemical properties.

## 1. Introduction

1,2,4-Triazines are a large and promising group of nitrogen-containing heterocyclic compounds. Considerable attention has been paid to the synthesis and study of the properties of monocyclic and fused 1,2,4-triazines as induced by a broad range of bioactivities. 1,2,4-Triazine-based compounds and their fused congeners have anticancer, antimicrobial, antifungal, anti-inflammatory, antimalarial, and antiviral properties [1,2,3,4]. Since 1,2,4-triazines fused with five-membered heterocycles are considered bio-isosteres with a purine core, these derivatives are the focus of drug design [5,6,7,8,9,10,11]. However, 1,2,4-triazines possessing specific photophysical properties have seldom been used in optoelectrical research. Only a few 1,2,4-triazine derivatives have been employed as building blocks for organic photoactive materials, particularly organic light-emitting diodes [12,13,14,15,16,17,18]. However, their closed nitrogen congeners (such as triazoles, pyrimidines, and *s*-triazines) are an inexhaustible source of numerous photoactive substances for various applications in optoelectronics and luminescent materials [19,20,21,22,23].

### 1.1. Previous Works

**Scheme 1:**
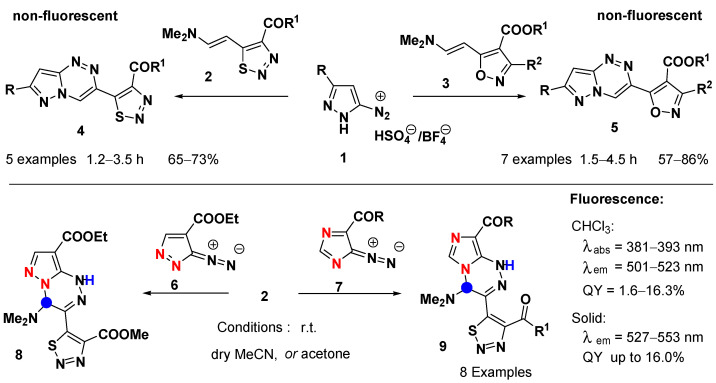
Strategies for aromatic azolo[5,1-*c*][1,2,4]triazines **4**, **5** and non-aromatic DATs **8**, **9** via the reactions of azole diazonium salts **1** or diazoazoles **6** and **7** with heterocyclic enamines.

The reaction of azole diazonium salts **1** [24] with heterocyclic enamines **2** and **3** leads to azolo[5,1-*c*][1,2,4]triazines **4** and **5** [25] (Scheme 1). Analogous azolotriazines were synthesized previously by using enamines, obtained from various secondary linear *N*,*N*-dialkylamines, and cyclic amines—pyrrolidine, piperidine, morpholine [26,27,28,29,30,31,32,33,34,35]. We have found that diazoazoles **6** and **7** [36,37] react with enamines **2** to produce the DATs **8** and **9** [38] (Scheme 1). Thus, this reaction is managed by the diazo reagent structure and could be a source of both aromatic azolo[5,1-*c*][1,2,4]triazines **4** and **5** and non-aromatic DATs **8** and **9**. Moreover, this finding points to a new approach to DATs, previously considered only as intermediates in the pathway to aromatic analogs.

To describe the mechanism of heterocyclic backbone formation, several alternatives have been considered. Many comprehensive experimental and theoretical studies have revealed suitable pathways [26,27,28,29,30,31,32,33,34,35]. However, neither experimental results [30,32] nor quantum chemical calculations [26] have given a clear answer to the question of the reaction mechanism and the role of the diazo reagent.

We detected the reaction intermediate using ^1^H NMR experiments before isolating and characterizing it. This finding introduced some certainty into the question of the reaction mechanism in favor of 1,3-dipolar cycloaddition [38]. This highly efficient process is characterized by mild conditions, high yields, availability, the variability of starting reagents, and the easy isolation of the products. The listed advantages are very important, since DAT **9** is fluorescent and can be considered as a new candidate in the set of fluorosensors due to the sensitivity of its photophysical characteristics to protic or polar solvents.

### 1.2. This Work

**Scheme 2:**
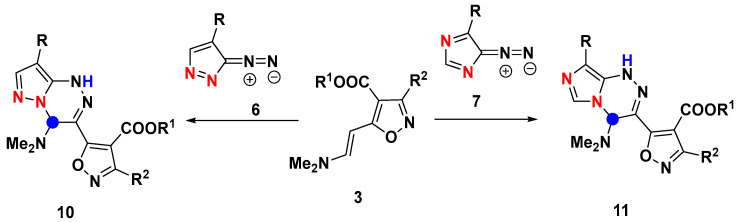
Design of new DATs–NMe_2_ **10** and **11** isoxazole at C4 atom of heterocyclic core.

The aim of this work was to expand the scope of the reaction between diazoazoles and enamines by synthesizing new DATs **10** and **11** (Scheme 2), bearing an isoxazole heterocycle at position C3 instead of a thiadiazole ring. This study was carried out to synthesize and investigate their photophysical properties (Scheme 2).

The choice of isoxazole was not accidental. Isoxazole rings are very popular in medicinal chemistry [39,40,41,42,43] and it is a structural part of many photoactive compounds [44,45]. The isoxazole electronic structure differs from 1,2,3-thiadiazole, exhibiting more electron saturation at the aromatic cycle. This enables it to change the optical characteristics and sensitivity of DATs depending on the microenvironment [45,46].

## 2. Results and Discussion

### 2.1. Chemistry

**Scheme 3:**
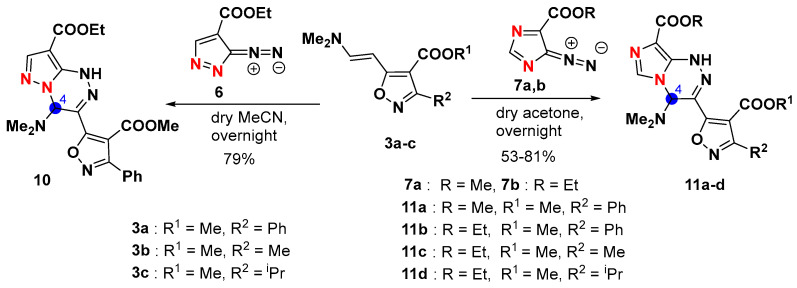
Synthesis of DATs–NMe_2_ **10** and **11a**–**d**.

The reaction of 5-diazoazoles **6** and **7a**,**b** and isoxazol-5-yl enamines **3a**–**d** led to the formation of non-aromatic DAT–NMe_2_ **10** and **11a**–**d** in moderate to good yields (Scheme 3) [38]. The process was carried out in dry aprotic solvents at room temperature for 12–24 h. The starting enamines **3a**–**d** [47] and diazoazoles **6** and **7a**,**b** [36] were obtained by procedures described previously. 

The isolated compounds **10** and **11a**–**d** were characterized by ^1^H, ^13^C (BB) and HMBC NMR spectra, and by HRESMS and XRD (Supplementary Materials). The ^1^H NMR spectra of DATs–NMe_2_ **10** and **11a**–**d** contain the signals of all proton-containing groups (Figures S1–S5). The most important signal of the ^1^H NMR spectra of DATs–NMe_2_ **10** and **11a**–**d** is the C4H triazine ring proton signal, which was recorded at 6.26–6.48 ppm and shifted upfield at 3–4 ppm compared to triazines **4** and **5**. The ^13^C NMR spectra of derivatives **10** and **11a**–**d** contain a signal of C4 at 67.7–70.1 ppm, in agreement with their sp^3^ hybrid state. The NH proton displayed a broad singlet at 9.77–9.78 ppm (for DATs–NMe_2_ **11a**,**c**,**d** in CDCl_3_) and 11.98 and 12.10 ppm (for DATs–NMe_2_ **10** and **11b** in DMSO-*d*_6_), which disappeared when CD_3_COOD was added.

**Figure 1:**
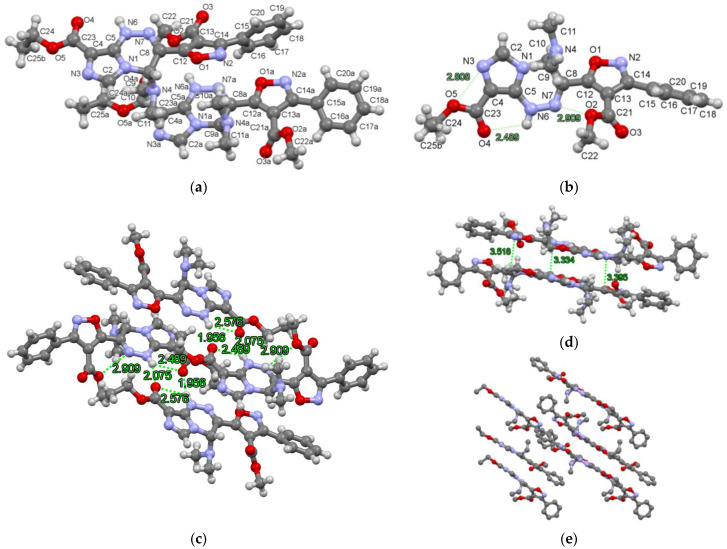
The molecular structure of DAT–NMe_2_ **11b** according to X-ray diffraction data: (**a**) unit of DAT–NMe_2_ **11b** including two molecules; (**b**) front view with intramolecular hydrogen bonding (dotted lines) (RN4H–O3 = 2.302 Å; RN5H–O1 = 2.968 Å); (**c**) location of neighbored molecules and intermolecular noncovalent bonds (R_VdW_(O–H) = 2.5 Å, R_VdW_(N–H) = 2.6 Å, R_VdW_(N–O) = 3.35 Å, R_VdW_(N–O) = 3.25 Å); (**d**) arrangement of molecules in two parallel sheets; (**e**) partial packing diagram along the crystallographic axis a. Hydrogen atoms omitted for clarity.

To refine the molecular structure, DAT–NMe_2_ **11b** was studied via single-crystal X-ray diffraction analysis. Single crystals of DAT–NMe_2_ **11b** were grown from their diluted solutions in acetone via the slow evaporation of the solvent. The molecular structures of the compound are shown in Figure 1 and Figure S1. Compound **11b** crystallizes into the centrosymmetric triclinic space group P-1. The asymmetric unit contains two independent molecules, the structure of which is unequal both in bond length and angle values due to the rotation around the C8–C12 bond, the linked bicyclic core, and the isoxazole ring (Figure 1a). Selected bond lengths and torsion angles are presented in Table S1. The structures in Figure 1 distinctly demonstrate different deviations of the isoxazole ring in different molecules from the bicyclic core, as well as deviations of the phenyl ring plane from the isoxazole ring. The bond lengths are slightly different in the two neighboring molecules; however, their values are lower than the standard ones, indicating that there is a conjugation within the molecule structure. Using a Mercury software package, several short intramolecular contacts were found in the crystal of compound **11b** (Figure 1b). The hydrogen bond involving the atom O4 and N6H hydrogen (*l* = 2.489 Å, R_VdW_ = 2.6 Å) and the two noncovalent bonds formed by atoms O2–N7 (*l* = 2.909 Å, R_VdW_ = 2.9 Å) and N3–O5 (*l* = 2.808 Å, R_VdW_ = 2.9 Å) enhance the rigidity of the molecule skeleton (Figure 1b). A packing structure is formed by the set of parallel layers (Figure 1d,e). The neighboring molecules in the layer form five bonds that connect them together firmly (Figure 1c). The strongest specific bond is the NH–O hydrogen bond, with a length of 1.905 Å. Table S1 shows that the bicyclic core and isoxazole ring in molecule A of the DAT–NMe_2_ **11b** structure are planar, while the phenyl ring deviates at 27.7(5)°. The other molecule demonstrates deviations between the isoxazole and imidazotriazine plains at 57.0°, while the phenyl ring rejects the isoxazole at 154.5(5)°.

The distance between layers in the packing was 3.334–3.395 Å (Figure 1d). NMe_2_ and alkoxycarbonyl groups, as well as phenyl rings, push the layers apart, preventing the formation of stronger contacts. Therefore, the planes of the cyclic fragments can only achieve the partial perturbation of orbitals in parallel layers, preventing strong π⋯π stacking. These spatial peculiarities lead to the formation of a specific 3D structure (Figure 1e and Figure S1).

**Scheme 4:**
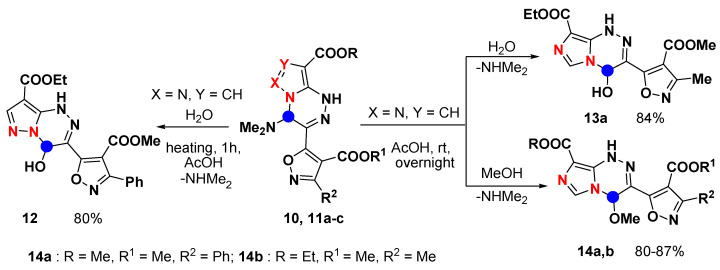
Synthesis of DATs–OH **12**, **13a** and DATs–OMe **14a**,**b**.

It was found that the stirring of the DAT–NMe_2_ **10**, **11a**–**c** in MeOH or in a wet solvent induces the replacement of the NMe_2_ group with the MeO or OH group, respectively, and the formation of DATs–OH **12** and **13a** and DATs–OMe **14a**,**b** with a good yield (Scheme 4). The reaction proceeds better in the presence of acetic acid or when being heated.

**Scheme 5:**
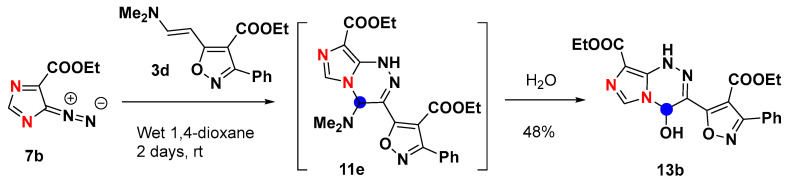
One-pot synthesis of DAT–OH **13b**.

The ‘one-pot’ cycloaddition of 5-diazoimidazole **7b** to enamine **3d** in wet 1,4-dioxane and further hydrolysis allows us to obtain DAT–OH **13b** in a 48% yield (Scheme 5). Thus, this technique can be used for the straightforward synthesis of 4-OH and 4-OMe derivatives of DATs.

The prepared DATs–OH **12**, **13a**,**b** and DATs–OMe **14a**,**b** were characterized by ^1^H, ^13^C NMR (including HSQC and HMBC for DAT **13b**) (Figures S6–S10), HRESMS, and XRD data. The main sets of signals observed in the ^1^H and ^13^C spectra are similar to the spectra for DAT–NMe_2_ **10** and **11**. The exception is the appearance of the MeO group’s singlet at 3.14–3.22 ppm for compounds **14a**,**b** and a doublet of OH (at 6.75–6.96 ppm) and C4H (at 7.71 and 7.80 ppm) with ^4^*J* = 7.8–8.5 Hz instead of a NMe_2_ proton singlet. Mass spectra (HRESMS) analysis showed the correct ion peaks [M+H]^+^ suggested by the molecular formulas.

**Figure 2:**
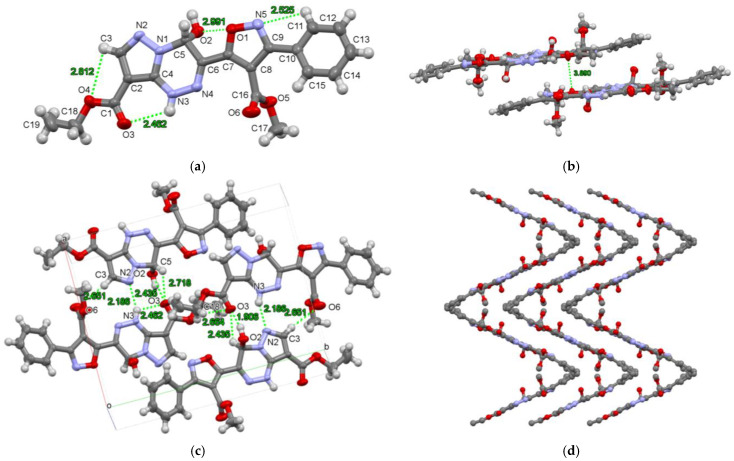
The molecular structure of DAT–OH **13b** according to X-ray diffraction data level: (**a**) front view with intramolecular bonds; (**b**) arrangement of molecules in two parallel sheets; (**c**) layer of neighboring molecules with intermolecular noncovalent bonds (R_VdW_(O–H) = 2.5 Å, R_VdW_(N–H) = 2.6 Å, R_VdW_(N–O) = 3.35 Å, R_VdW_(N–O) = 3.25 Å); (**d**) partial packing diagram along the crystallographic axes a (**d**). Hydrogen atoms omitted for clarity.

The structure of DAT–OH **13b** was confirmed by the XRD data from a single crystal growing in ethanol (Figure 2). The structure completely agrees with the physical and spectral data. The unit includes one molecule with four intramolecular noncovalent bonds (Figure 2). This makes the compound structure flatter than molecule **11b** and shows a more ordered structure in the package (Figure 2b). This molecular architecture is supported by the many intermolecular hydrogen bonds between the two neighboring molecules in the layers (Figure 2c), taking a zigzag shape (Figure 2d).

**Scheme 6:**
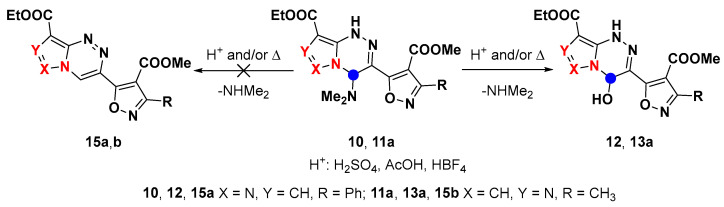
Transformation of DATs–NMe_2_ **10** and **11a** in the presence of acids and on heating.

The attempts to aromatize DATs–NMe_2_ **10**, **11a** to azolo[5,1-*c*][1,2,4]triazines, as occurred in the reaction of azole diazonium salts with the enamines [25], by the addition of glacial AcOH, as well as catalytic amounts of H_2_SO_4_ or equimolar amounts of HBF_4_, failed. As a result, hydrolysis to the corresponding non-aromatic of DAT–OH **12**, **13a** proceeded (Scheme 6).

### 2.2. Photophysical Properties of DATs

#### 2.2.1. Spectroscopic Properties in a Chloroform Solution

The structures of the synthesized compound do not have an extended π-conjugated framework because the two heteroaromatic cycles (imidazole or pyrazole) are divided by a non-aromatic dihydro-1,2,4-triazine cycle and linked via a σ bond with another heteroaromatic (isoxazole). The lateral substituents are both electron-withdrawing (COOR) and electron-donating groups (NMe_2_, OMe, OH at C4 atom of the dihydrotriazine ring and the Me group in isoxazole). The XRD data revealed the complicated character of the 3D structure, where the isoxazole rings of some molecules of DAT–NMe_2_ **11b** can form a conjugation with the bicyclic core (Figure 1). However, other molecules are strongly repelled by this ring from the central core’s plane and therefore have slight conjugation within the molecule. The abundance of heteroatoms in the cyclic fragments and lateral substitutions with mobile electrons, and the presence of NH and OH groups, can provide DATs with specific photophysical properties, such as sensitivity to the microenvironment and the ability to induce differences in the investigated set of compounds.

**Figure 3:**
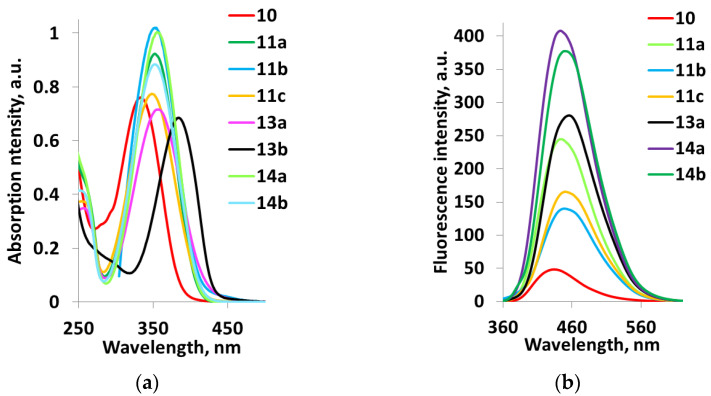
(**a**) Absorption and (**b**) emission spectra of DATs **10**, **11a**–**c**, **12a**,**b**, **13a**,**b** solutions in CHCl_3_ (*c* = 5 × 10^−5^ M for adsorption and *c* = 5 × 10^−6^ M for emission).

DATs are soluble in organic solvents. Only hydroxy derivative **13a** shows limited solubility in non-polar solvents, while DAT–OH **13b** is insoluble in toluene, in contrast to its NMe_2_ or OMe congeners. DAT solutions are colorless; however, they exhibit blue or blue-green fluorescence upon UV irradiation. The absorption spectra of DATs–NMe_2_ **10** and **11a**–**c**,**e**, DATs–OH **12**, **13a**,**b**, and DATs–OMe **14a**,**b** show the same absorption spectra profile, with one band in the 321–384 nm range (Table 1 and Figure 3a). Emission maxima of DATs–NMe_2_ are in the range of 433 to 488 nm (Table 1 and Figure 3b). DAT–NMe_2_ **10** with pyrazole in the bicyclic core displays significant hypsochromic shifts in the absorption and emission maxima (1205–3899 cm^−1^ and 1863–2603 cm^−1^, respectively) (Figure S3).

The DAT quantum yields (QYs) vary from 3.4 to 33.3%. DAT–NMe_2_ **10** shows the lowest QY in a chloroform solution, while DAT–OH **13b** is not fluorescent at all (Figure S3). It is worth emphasizing the significant Stokes shift of DATs **11a**–**d** and **13** (Table 1), demonstrating the large differences between the ground (GS) and excited (ES) states in the electronic structure and revealing a partial intramolecular charge transfer (ICT) upon vertical excitation. The phenyl ring at the C5 isoxazole does not increase the ICT, since the maxima absorption and emission are closed to the analogous structure, bearing a Me substituent. This means that the rotation of this structure fragment prevents it from participating in the molecule’s conjugation channel. These conclusions are in agreement with the XRD data analysis (*vide supra*).

It should be mentioned that the DATs **10**–**14** displayed significant hypsochromic shifts in the absorption and emission maxima in comparison with the maxima of similar derivatives **9** (Scheme 1), bearing a thiadiazole moiety at the C4 atom of the azolotriazine core [38]. However, the DATs **9** molar absorption coefficients are 1.4 fold and QYs 2.0–3.8 fold lower than DATs’ **10**–**14** absorption and emission intensity characteristics. Thus, this position of the fluorophore molecule is effective for the tuning of its electronic state and photophysical properties [48].

**Table 1:** Photophysical characteristics of DATs **10**, **11a**–**c**,**e**, **13a**,**b**, and **14a**,**b** in CHCl_3_ (*c* = 5 × 10^−5^ M for adsorption and *c* = 5 × 10^−6^ M for emission).

Entry	Compd.	λ_max_, nm	ε, M^−1^·cm^−1^	λ_em_, nm	QY *^a^*, %	Stokes Shift, nm/cm^−1^
1	**10**	334	15,200	433	6.1	99/6845
2	**11a**	352	18,500	471	19.0	119/7178
3	**11b**	350	20,400	475	12.3	125/7519
4	**11c**	348	15,500	473	16.8	125/7594
5	**13a**	357	13,200	488	29	131/7519
6	**13b**	384	13,700	–	–	–
7	**14a**	355	20,100	473	33.3	118/7027
8	**14b**	352	17,700	477	31.3	125/7445

*^a^*—Relative quantum yield [49], determined relative to the standard (quinine sulfate solution *c* = 5 × 10^−5^ M in 0.1 M H_2_SO_4_, Φ_F_ = 54.0%).

The fluorescence lifetimes of the DATs were measured in chloroform at room temperature using the time-correlated single photon counting technique (Table 2). All decay (Figure S4) can be fitted well with the double exponential decay function, exhibiting the coexistence of two fluorescent species in the solvent. DAT–OH **13a** demonstrated the longest fluorescence lifetime, followed by DAT–OMe **14b** and DAT–NMe_2_ **11c**. DAT–NMe_2_ **10** had the shortest fluorescence lifetime and the highest velocity of non-radiative energy dissipation.

#### 2.2.2. The Solvatochromic Behaviour of DATs

**Figure 4:**
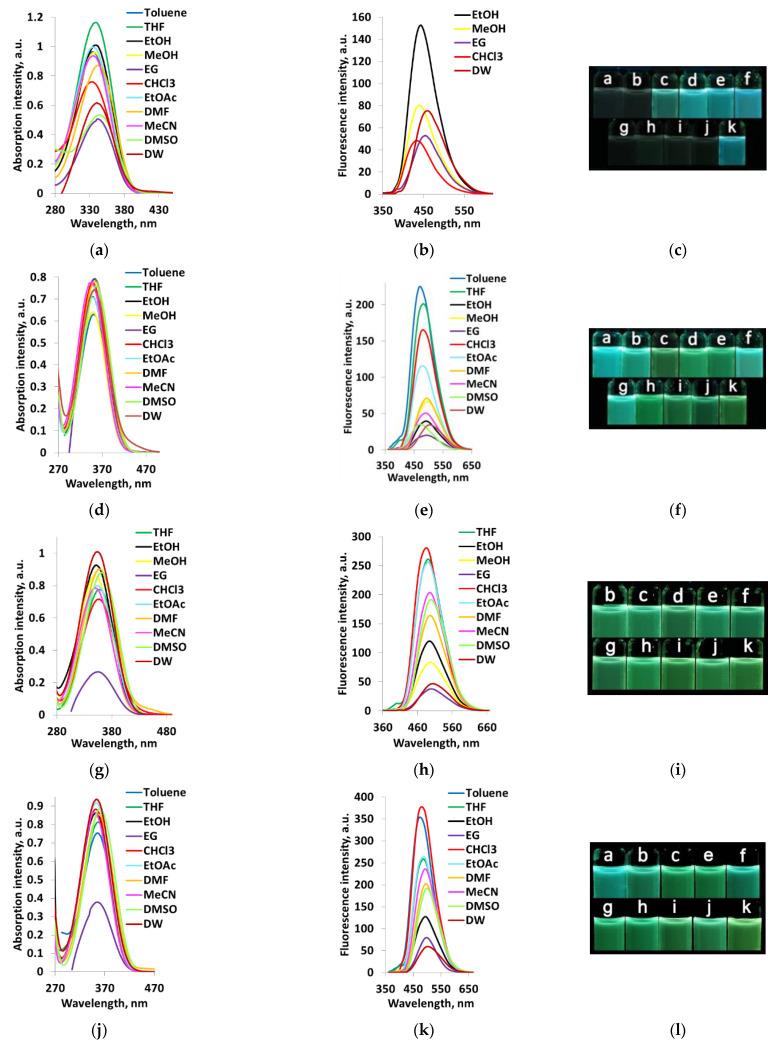
Absorption (**a**,**d**,**g**,**j**) and emission (**b**,**e**,**h**,**k**) spectra of solutions of compounds **10** (**a**,**b**), **11c** (**d**,**e**), **13a** (**g**,**h**), and **14b** (**j**,**k**) in various solvents (**c**,**f**,**i**,**l**); photographs of solutions of compounds **10** (**c**), **11c** (**f**), **13a** (**i**), and **14b** (**l**) under UV irradiation (λ = 365 nm). Solvents: a—toluene, b—THF, c—EtOH, d—MeOH, e—EG, f—CHCl_3_, g—EtOAc, h—MeCN, i—DMF, j—DMSO, k—DMSO–H_2_O (*v*/*v*, 1:9) (c = 5 × 10^−5^ M).

The UV–Vis absorption and fluorescence spectra of DATs **10** and **11** in 10 solvents are shown in Figure 4 and Table S4. The obtained results demonstrate the solvent’s effect on the optical properties of the investigated compounds. The absorption maxima change within a small range (5–7 nm), while the molar extinction coefficient diminishes significantly, passing from toluene to the DMSO. The most unexpected result is the difference in the photophysical properties of DAT–NMe_2_ **10** and DAT–NMe_2_ **11c**, **13a**, and **14b**. DAT–NMe_2_ **10** exhibited emissions only in protic solvents (EtOH, MeOH, ethylene glycol (EG)) and chloroform. This clearly indicates that solvents capable of forming specific interactions with the dye can promote fluorescence. Moreover, the greater the redshift of the emission maximum, the stronger these interactions are. Therefore, the largest shift is observed in the DMSO–water mixture (*v*/*v*, 1/9), while the smallest is registered in chloroform. The QY has rather random values, ranging from 6 to 15%. Ethylene glycol’s influence is not only as a protic solvent, but also as a viscous one. Therefore, the QY increased compared to methanol 2.5-fold.

The absorption maxima are weakly dependent on the nature of the solvent, unlike DAT–NMe_2_ **10**. DATs **11c**, **13a**, and **14b** obviously exhibit positive solvato(fluoro)chromism and a red shift in the polar solvents. For example, in DMSO, these displacements are 1156, 706, and 1057 cm^−1^ for DATs **11c**, **13a**, **14b**, respectively, passing from non-polar toluene, or THF for DAT **13a**. The best QYs were registered in non-polar toluene for **11c** and **14b** and THF for compound **13a**. In contrast to DAT–NMe_2_ **10**, the QYs of the compounds **11c**, **13a**, and **14b** decrease in alcohol in comparison with both polar and non-polar solvents. The Stokes shift is large, ranging within 7295–8783 cm^−1^ (with the maximum value provided by acetonitrile for DAT–NMe_2_ **11c**).

**Figure 5:**
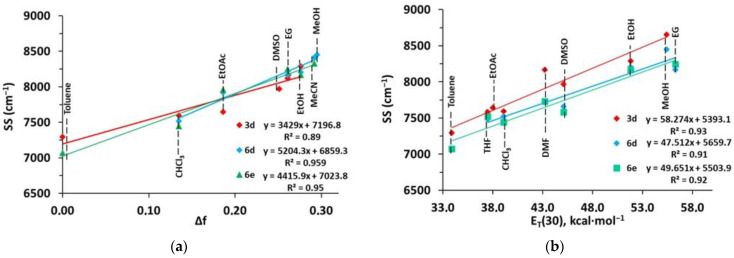
(**a**) The Lippert–Mataga plot showing the Stokes shift (SS) against solvent polarizability (Δf); (**b**) the Dimroth–Reichardt plot showing the Stokes shift (SS) against the ET(30) parameter of DATs **11c**, **13a**, and **14b**.

The obtained results demonstrate the significant influence of the nature of the solvent used on the DATs’ fluorescence characteristics. Therefore, the solvatochromic behavior of DATs **11c**, **13a**, and **14b** was analyzed using Lippert–Mataga [49,50,51] (Equation (S1)) and Dimroth–Reichardt (E_T_(30)), (Equation (S6)) [52,53,54,55] solvent polarity plots (Figure 5). The Lippert–Mataga Equation (S1) is based on the correlation of the energy difference between the ground and excited states (Stokes shift) and the solvent’s orientation polarizability (∆f). This expression of the Stokes shift only takes into account dipole–dipole interaction. The solution’s polarizability and the influence of specific interactions are neglected.

The correlation coefficients obtained from linear correlations (R^2^) were estimated using the maximum number of solvents with the aim of finding the best linearity. The values deviating from linearity were subtracted from the plots. As a result, good linearity (R^2^ = 0.89–0.96) was obtained with the exception of MeCN and EG for DAT–NMe_2_ **11c** and THF and DMF for **13a** and **14b**. The Lippert–Mataga plot shows good linearity, indicating an increase in the solvatochromic shift as the solvent polarity increases (Figure 5a). The positive slope obtained for each of the DATs under consideration exhibits a larger excited-state dipole moment than the ground-state dipole moment [49]. It is suggested that the dielectric interaction of the surrounding solvents is very responsible for the observed spectral shifts of the studied fluorophores. The slope values demonstrate that DAT–NMe_2_ **11c** is significantly less dependent on the solvent orientation polarizability (∆f) than DATs **13a** and **14b**. Therefore, a dipole–dipole interaction between the solute and the solvents for compound **11c** is less important than for compounds **14b** and **13a**.

The advantage of the Dimroth–Reichardt method over the Lippert–Mataga method is the incorporation of both the solvent polarity and hydrogen bonding in the solvent parameter. The Dimroth–Reihardt plot of the Stokes shift for DAT–NMe_2_ **11c** via the ET(30) solvent polarity parameter (Figure 5b) displays a better correlation (R^2^ = 0.93) than that proposed by the Lippert–Mataga equation (excepting MeCN and EG). The Dimroth–Reichardt plots for DATs **13a** and **14b** showed a slight decrease in the linearity obtained, with the exception of MeCN and EtOAc (R^2^ = 0.91 and 0.92, respectively). Moreover, the slope of DAT–NMe_2_ **11c** is higher than those of DATs **13a** and **14b**, which were obtained according to the Dimroth–Reichardt correlation. This result confirms the conclusions stated on the basis of the Lippert–Mataga equation. Hydrogen bonding is very important for the solvatochromic behaviour of DAT–NMe_2_ **11c**. Compound **11c** is more sensitive to the specific interaction with solvents than **13a** and **14b**.

#### 2.2.3. Spectroscopic Properties in a Solid State

**Figure 6:**
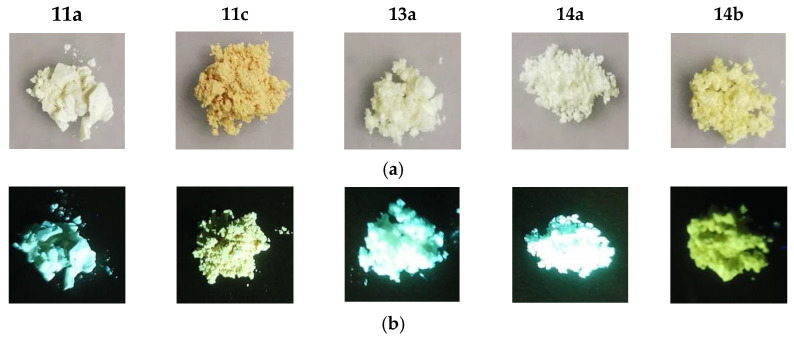
Photographs of DATs **11a**,**c**, **13a**, and **14a**,**b** in a solid state under (**a**) daylight and (**b**) UV radiation with a hand-held UV lamp at an emission wavelength of 365 nm.

The fluorescence of the DAT powders was measured using an integrating sphere. The absolute QYs of the investigated DATs are in the range of 9.8 to 98.7% (Figure 6 and Table 3): most of them are higher than the QY in CHCl_3_ (1.2–3.0-fold). The maximum emission wavelengths in the solid state are very close to those obtained in chloroform for DAT **11a**, **13a**, and **14a**: they are redshifted by 1300 cm^−1^ and 722 cm^−1^ for DAT–NMe_2_ **11c** and DAT–OMe **14b**, respectively. Compounds **11c** and **14b** exhibited the lowest QYs among the compounds demonstrating fluorescence in a solid state. In addition, their structure has the same substituents, a decorated heterocycle ring in the molecular structure: they are differentiated only by NMe_2_ and OMe groups on the C4 atom.

#### 2.2.4. Study of DATs’ Aggregation-Induced Emission (AIE) and Aggregation-Induced Enhancement (AIEE)

The behavior of a fluorophore in aquatic environments plays an important role in determining the scope of the application, including such important fields as biology, medicine, and ecology [56,57,58,59,60]. Neutral organic dyes and fluorophores are poorly soluble in water and aqueous media. When using binary mixtures of an organic solvent with water, they are able to form suspensions. This process can cause two opposite photophysical behaviors: aggregation-caused fluorescence quenching (ACQ) or aggregation-induced emission (AIE). The reason behind ACQ is the increased redistribution of energy between the excited and unexcited molecules and the emergence of new channels for the loss of excitation energy during the aggregation. However, some compounds have a specific molecular architecture that prevents this energy loss: this is due to the presence of structural elements that induce the restriction of intramolecular motion (RIM) [56,57,58]. This can include bulky groups, structural peculiarities that decrease intermolecular interaction, the donors and acceptors of hydrogen bonds, and the insertion of molecules of solvents into the molecular packing structure.

Preliminary experiments in a DMSO–H_2_O mixture (*v*/*v*, 1/9) (Table 4) demonstrate the appearance of DAT **10** emissions in a binary DMSO–H_2_O mixture. DATs–NMe_2_ **11a**,**c** keep the emissions at approximately the same level in DMSO, while DATs **13a** and **14a**,**b** show a 4.8–5.0-fold decrease in emission intensity in the DMSO–H_2_O mixture. The DAT maxima absorption and emissions are slightly redshifted to 320–563 cm^−1^ and 201–405 cm^−1^, respectively.

**Figure 7:**
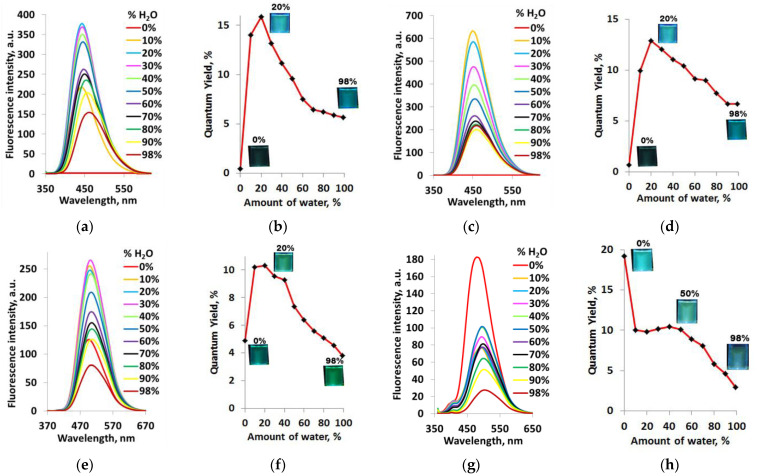
(**a**,**c**,**e**,**g**) Fluorescence spectra of DATs (**a**,**c**) **10** and (**e**,**g**) **11c** (c = 1 × 10**^−^**^5^ M) in the (**a**,**e**) DMSO–H_2_O and (**c**,**g**) THF–H_2_O mixtures with gradually changing water content. (**b**,**d**,**f**,**h**) Plot of the emission intensity (QY) of DATs (**b**,**d**) 10 and (**f**,**h**) **11c** versus a water fraction from 0 to 100% in (**b**,**f**) a DMSO–H_2_O and (**d**,**h**) a THF–H_2_O mixture. Inserts: photographs of DATs–NMe_2_ **10** and **11c** solutions in DMSO–H_2_O and THF–H_2_O mixtures (*v*/*v*, 1:9).

To reveal the aggregation-induced emission properties of DATs–NMe_2_ **10** and **11c**, the fluorescence intensities at maximum emission were recorded in a mixture of DMSO–H_2_O and THF–H_2_O with different water content (Figure 7, Figures S5 and S6 and Tables S5–S8).

Thus, a sample of DAT–NMe_2_ **10** in a mixture of DMSO–H_2_O and THF–H_2_O became fluorescent when 20% of water was added. The fluorescence intensity decreases, but even at the ratio of DMSO–H_2_O and THF–H_2_O (*v*/*v*, 99:1), the QY holds at 6%. DAT–NMe_2_ **11c** had the opposite behavior, as the QY in DMSO–H_2_O showed a sharp increase as the water content increased up to 10%; then, there was a gradual decrease to 6%. In the THF–H_2_O mixture, the QY of DAT–NMe_2_ **11c** exhibited a sharp decrease (2-fold), and then the compounds held the fluorescence intensity at this level up to 50% water content and at the end slowly decreased. This research clearly shows that DAT–NMe_2_ **10** is a new AIE gene, has a strong effect, and can be used in aquatic environments.

## 3. Materials and Methods

### 3.1. Chemistry

^1^H and ^13^C NMR spectra were recorded on a Bruker Avance II 400 (400 and 100 MHz, respectively) spectrometer or a Bruker Avance NEO 600 (600 and 150 MHz, respectively) spectrometer, equipped with the broadband gradient Prodigy Cryoprobe and using DMSO-*d*_6_ or CDCl_3_ as the solvent and TMS as an internal standard. ^1^H NMR data are reported as the chemical shift in parts per million, multiplicity (s, singlet; br. s, broadened singlet; d, doublet; t, triplet; q, quartet; m, multiplet), coupling constant in hertz, and number of protons. The concerted application of ^1^H–^13^C 2D heteronuclear experiments HSQC and HMBC was used for distinguishing carbon and proton resonances. Mass spectra were recorded with a Shimadzu GCMS-QP 2010 “Ultra” (Kyoto, Japan) mass spectrometer using the electron impact (EI) ionization technique (40–200 °C, 70 eV). Spectra of exact mass were acquired on a quadrupole orthogonal acceleration time-of-flight mass spectrometer (Synapt G2 HDMS, Waters, Milford, MA, USA). Samples were infused at 3 uL/min and spectra were obtained in positive (or negative) ionization mode with a resolution of 15,000 (FWHM), using leucine enkephalin as a lock mass. The abbreviation [M]^+^ refers to the molecular ion. Elemental analysis was performed on a PerkinElmer 2400 II CHNS-analyzer. All melting points were determined with a Stuart SMP3 apparatus.

Commercial reagents were obtained from Sigma-Aldrich, Acros Organics, or Alfa Aesar and used without any preprocessing. All workup and purification procedures were carried out using analytical-grade solvents.

The starting diazopyrazole **6** [61], diazoimidazoles **7a**,**b** [62,63], and enamines **3a**–**d** [47] were obtained as previously described.

Ethyl (*N*,*N*-dimethylamino)-3-[4-(methoxycarbonyl)-3-phenyl-1,2-oxazol-5-yl]-1,4-dihydropyrazolo[5,1-*c*][1,2,4]triazine-8-carboxylate (**10**): A mixture of diazopyrazole **6** (166 mg, 1.0 mmol), enamine **3a** (272 mg, 1.0 mmol), and dry acetonitrile (5 mL) was stirred at room temperature for 12 h. The solvent was evaporated *in vacuum*, and the residue was purified by column chromatography on silica gel (eluent—DCM/acetone = 5:1). Yield 346 mg (79%), white solid, mp 158–160 °C. ^1^H NMR (400 MHz, DMSO-*d*_6_) δ 12.10 (1H, br. s, NH), 7.93 (1H, s, H), 7.65 (2H, dd, *J* = 7.4 Hz, *J* = 1.9 Hz, H), 7.54–7.57 (3H, m, H), 6.30 (1H, s, H), 4.28 (2H, q, *J* = 7.1 Hz, OCH_2_CH_3_), 3.80 (3H, s, OCH_3_), 2.18 (6H, s, N(CH_3_)_2_), 1.31 (3H, t, *J* = 7.1 Hz, OCH_2_CH_3_). ^13^C NMR (101 MHz, DMSO-*d*_6_) δ 166.2, 162.2, 161.7, 160.8, 140.2, 139.5, 130.3, 128.7, 128.1, 128.0, 127.1, 109.5, 94.5, 70.1, 59.5, 52.5, 39.5, 14.3. HRMS (ESI^+^), *m*/*z* calcd. for C_21_H_22_N_6_O_5_ [M+H]^+^ 439.1724, found 439.1717. Found, %: C 57.27, H 5.24, N 19.29. C_21_H_22_N_6_O_5_. Calculated, %: C 57.53, H 5.06, N 19.17.

Methyl (*N*,*N*-dimethylamino)-3-[4-(methoxycarbonyl)-3-phenyl-1,2-oxazol-5-yl]-1,4-dihydroimidazo[5,1-*c*][1,2,4]triazine-8-carboxylate (**11a**): Compound **11a** was synthesized by the same procedure as **10** using diazoimidazole **7a** (166 mg, 1.0 mmol). Yield 343 mg (81%), yellow solid, mp 197–200 °C. ^1^H NMR (400 MHz, CDCl_3_) δ 9.78 (1H, br. s, NH), 7.67 (2H, dd, *J* = 6.8 Hz, H), 7.47–7.51 (3H, m, H), 7.39 (1H, s, H), 6.30 (1H, s, H), 3.96 (3H, s, OCH_3_), 3.81 (3H, s, OCH_3_), 2.28 (6H, s, N(CH_3_)_2_). ^13^C NMR (101 MHz, CDCl_3_) δ 165.9, 163.9, 162.9, 161.9, 135.2, 130.2, 129.5, 128.6, 128.5, 127.7, 127.3, 110.9, 110.3, 69.0, 52.5, 51.8, 40.0. HRMS (ESI^+^), *m*/*z* calcd. for C_20_H_20_N_6_O_5_ [M+H]^+^ 425.1568, found 425.1579. Found, %: C 56.68, H 4.84, N 19.62. C_20_H_20_N_6_O_5_. Calculated, %: C 56.60, H 4.75, N 19.80.

Ethyl (*N*,*N*-dimethylamino)-3-[4-(methoxycarbonyl)-3-phenyl-1,2-oxazol-5-yl]-1,4-dihydroimidazo[5,1-*c*][1,2,4]triazine-8-carboxylate (**11b**): Compound **11b** was synthesized by the same procedure as **10** using diazoimidazole **7b** (166 mg, 1.0 mmol). Yield 311 mg (71%), yellow solid, mp 185–187 °C. ^1^H NMR (400 MHz, DMSO-*d*_6_) δ 11.98 (1H, br. s, NH), 7.70 (1H, s, H), 7.65 (2H, dd, *J* = 7.4 Hz, *J* = 1.9 Hz, H), 7.54–7.56 (3H, m, H), 6.38 (1H, s, H), 4.28 (2H, q, *J* = 7.0 Hz, OCH_2_CH_3_), 3.80 (3H, s, OCH_3_), 2.12 (6H, s, N(CH_3_)_2_), 1.28 (3H, t, J = 7.0 Hz, OCH_2_CH_3_). ^13^C NMR (101 MHz, DMSO-*d*_6_) δ 166.3, 162.4, 162.0, 160.9, 134.3, 130.9, 130.5, 128.9, 128.0, 127.2, 126.0, 110.0, 109.2, 67.7, 59.4, 52.7, 39.6, 14.5. HRMS (ESI^+^), *m/z* calcd. for C_21_H_22_N_6_O_5_ [M+H]^+^ 439.1724, found 439.1728. Found, %: C 57.76, H 4.93, N 19.03. C_21_H_22_N_6_O_5_. Calculated, %: C 57.53, H 5.06, N 19.17.

Ethyl 4-(*N*,*N*-dimethylamino)-3-[4-(methoxycarbonyl)-3-methyl-1,2-oxazol-5-yl]-1,4-dihydroimidazo[5,1-*c*][1,2,4]triazine-8-carboxylate (**11c**): Compound **11c** was synthesized by the same procedure as **10** using diazoimidazole **7b** (166 mg, 1.0 mmol) and enamine **3b** (210 mg, 1.0 mmol). Yield 278 mg (74%), yellow solid, mp 140–142 °C. ^1^H NMR (400 MHz, CDCl_3_) δ 9.78 (1H, br. s, NH), 7.35 (1H, s, H), 6.41 (1H, s, H), 4.42 (2H, q, *J* = 7.0, OCH_2_CH_3_), 3.90 (3H, s, OCH_3_), 2.49 (3H, s, CH_3_), 2.23 (6H, s, N(CH_3_)_2_), 1.43 (3H, t, J = 7.0 Hz, OCH_2_CH_3_). ^13^C NMR (101 MHz, CDCl_3_) δ 168.5, 163.5, 162.3, 160.1, 135.1, 129.4, 127.8, 111.0, 110.3, 69.2, 60.8, 52.1, 40.0, 14.5, 11.9. HRMS (ESI^+^), *m*/*z* calcd. for C_16_H_20_N_6_O_5_ [M+H]^+^ 377.1345, found 377.1598. Found, %: C 51.19; H 5.11; N 22.52. C_16_H_20_N_6_O_5_. Calculated, %: C 51.06; H 5.36; N 22.33.

Ethyl 4-(*N*,*N*-dimethylamino)-3-[3-isopropyl-4-(metoxycarbonyl)isoxazol-5-yl]-1,4-dihydroimidazo[5,1-*c*][1,2,4]triazine-8-carboxylate (**11d**): Compound **11d** was synthesized by the same procedure as **10** using diazoimidazole **7b** (166 mg, 1.0 mmol) and enamine **3c** (238 mg, 1.0 mmol). Yield 214 mg (53%), yellow solid, mp 165–167 °C. ^1^H NMR (400 MHz, CDCl_3_) δ 9.77 (1H, br. s, NH), 7.35 (1H, s, H), 6.26 (1H, s, H), 4.43 (2H, q, *J* = 7.1 Hz, OCH_2_CH_3_), 3.88 (3H, s, OCH_3_), 3.34–3.41 (1H, m, CH(CH_3_)_2_), 2.23 (6H, s, N(CH_3_)_2_), 1.34–1.44 (9H, m, CH(CH_3_)_2_, OCH_2_CH_3_). ^13^C NMR (101 MHz, CDCl_3_) δ 168.1, 166.9, 163.6, 162.5, 135.2, 129.3, 127.9, 110.9, 109.6, 69.3, 60.7, 52.2, 40.0, 26.8, 21.1, 20.9, 14.5. HRMS (ESI^+^), *m*/*z* calcd. for C_18_H_24_N_6_O_5_ [M+H]^+^ 405.1808, found 405.1881. Found, %: C 53.22, H 6.15, N 20.93. C_18_H_24_N_6_O_5_. Calculated, %: C 53.46, H 5.98, N 20.78.

Ethyl 4-hydroxy-3-[4-(methoxycarbonyl)-3-phenyl-1,2-oxazol-5-yl]-1,4-dihydropyrazolo[5,1-*c*][1,2,4]triazine-8-carboxylate (**12**): Compound **10** (219 mg, 0.5 mmol) in the mixture of water and acetic acid (1:1.5 mL) was stirred at room temperature for 24 h. The reaction completion was checked by TLC; the precipitate was filtered off and purified by column chromatography on silica gel (eluent—CHCl_3_/EA = 4:1). Yield 165 mg (80%), white solid, mp 190–192 °C. ^1^H NMR (600 MHz, DMSO-*d*_6_) δ 12.39 (1H, br. s, NH), 7.95 (1H, s, H), 7.90 (1H, d, *J* = 7.8 Hz, OH), 7.65 (2H, dd, *J* = 8.0 Hz, *J* = 1.9 Hz, H), 7.54–7.58 (3H, m, H), 6.75 (1H, d, *J* = 7.8 Hz, H), 4.28 (2H, q, *J* = 7.1 Hz, OCH_2_CH_3_), 3.82 (3H, s, OCH_3_), 1.30 (3H, t, *J* = 7.1 Hz, OCH_2_CH_3_). ^13^C NMR (101 MHz, DMSO-*d*_6_) δ 166.0, 162.5, 161.7, 160.8, 140.9, 138.2, 130.6, 129.4, 129.0, 127.9, 127.2, 109.3, 94.9, 70.4, 59.6, 52.9, 14.5. HRMS (ESI^+^), *m*/*z* calcd. for C_19_H_17_N_5_O_6_ [M+H]^+^ 412.1252, found 412.1257. Found, %: C 55.64, H 4.22, N 16.86. C_19_H_17_N_5_O_6_. Calculated, %: C 55.48, H 4.17, N 17.02.

Ethyl 4-hydroxy-3-[4-(methoxycarbonyl)-3-methyl-1,2-oxazol-5-yl]-1,4-dihydroimidazo[5,1-*c*][1,2,4]triazine-8-carboxylate (**13a**): Compound **13a** was synthesized by the same procedure as **12** from compound **11c** (300 mg, 0.5 mmol). Yield 231 mg (84%), yellow solid, mp 215–218 °C. ^1^H NMR (600 MHz, DMSO-*d*_6_) δ 12.14 (1H, s, NH), 7.74 (1H, s, H), 7.71 (1H, d, *J* = 8.5 Hz, OH), 6.79 (1H, d, *J* = 8.5 Hz, H), 4.28 (2H, q, *J* = 7.1 Hz, OCH_2_CH_3_), 3.84 (3H, s, CH_3_), 2.40 (3H, s, CH_3_), 1.29 (3H, t, *J* = 7.1 Hz, OCH_2_CH_3_). ^13^C NMR (101 MHz, DMSO-*d*_6_) δ 166.7, 162.0, 161.8, 159.7, 132.5, 130.5, 127.5, 109.9, 109.6, 68.5, 59.3, 52.3, 14.6, 11.1. HRMS (ESI^+^), *m*/*z* calcd. for C_14_H_15_N_5_O_6_ [M+H]^+^ 350.1095, found 350.1087. Found, %: C 47.95, H 4.69, N 20.19. C_14_H_15_N_5_O_6_. Calculated, %: C 48.14, H 4.33, N 20.05.

Ethyl 3-[4-(ethoxycarbonyl)-3-phenyl-1,2-oxazol-5-yl]-4-hydroxy-1,4-dihydroimidazo[5,1-*c*][1,2,4]triazine-8-carboxylate (**13b**): A mixture of compound **7b** (166 mg, 1.0 mmol), enamine **3d** (286 mg, 1.0 mmol), and dioxane (2 mL) was stirred at room temperature for 2 days. The solvent was evaporated in vacuum and the residue was separated on the column with silica gel (eluent: chloroform—ethyl acetate = 4: 1). The fractions containing compound with R_f_ = 0.1 were collected. The solvent was evaporated *in vacuum* to dryness to give the product 13b. Yield 204 mg (48%), yellow solid, mp 205–207 °C. ^1^H NMR (400 MHz, DMSO-*d*_6_) δ 12.18 (1H, s, NH), 7.80 (1H, d, *J* = 8.0 Hz, OH), 7.75 (1H, s, H), 7.65 (2H, dd, *J* = 7.4 Hz, *J* = 1.9 Hz, H), 7.52–7.57 (3H, m, H), 6.80 (1H, d, *J* = 8.0 Hz, H), 4.24–4.33 (4H, m, 2OCH_2_CH_3_), 1.30 (3H, t, *J* = 7.1 Hz, OCH_2_CH_3_), 1.16 (3H, t, *J* = 7.1 Hz, OCH_2_CH_3_); ^13^C NMR (101 MHz, DMSO-*d*_6_) δ 165.7, 161.9, 161.8, 161.0, 132.7, 130.4, 130.3, 128.8, 128.1, 128.0, 127.3, 109.9, 109.2, 68.1, 61.6, 59.3, 14.5, 13.6. HRMS (ESI^+^), *m*/*z* calcd. for C_20_H_19_N_5_O_6_ [M+H]^+^ 426.1408, found 426.1411. Found, %: C 56.58, H 4.64, N 16.53. C_20_H_19_N_5_O_6_. Calculated, %: C 56.47, H 4.50, N 16.46.

Methyl 4-methoxy-3-[4-(methoxycarbonyl)-3-phenyl-1,2-oxazol-5-yl]-1,4-dihydroimidazo[5,1-*c*][1,2,4]triazine-8-carboxylate (**14a**): Compound **11a** (212 mg, 0.5 mmol) was refluxed in the mixture of methanol (10 mL) and acetic acid (1 mL) for 1 h. The reaction completion was checked by TLC, and the reaction mixture was evaporated *in vacuum* to dryness. The residue was purified by column chromatography on silica gel (eluent—CHCl_3_/EA = 4:1). Yield 164 mg (80%), yellow solid, mp 176–177 °C. ^1^H NMR (600 MHz, DMSO-*d*_6_) δ 12.46 (1H, br. s, NH), 7.99 (1H, s, H), 7.66 (2H, dd, *J* = 7.9 Hz, *J* = 1.6 Hz, H), 7.54–7.59 (3H, m, H), 6.85 (1H, s, H), 3.22 (3H, s, OCH_3_), 3.83 (3H, s, OCH_3_). 3.80 (3H, s, OCH_3_). ^13^C NMR (151 MHz, DMSO-*d*_6_) δ 165.3, 162.5, 162.1, 160.8, 133.0, 130.9, 130.6, 129.0, 127.9, 127.0, 124.7, 110.2, 108.9, 74.5, 54.5, 52.9, 50.9. HRMS (ESI^+^), *m*/*z* calcd. for C_19_H_17_N_5_O_6_ [M+H]^+^ 412.1252, found 412.1261. Found, %: C 55.10, H 3.86, N 17.36. C_19_H_17_N_5_O_6_. Calculated, %: C 55.48, H 4.17, N 17.02.

Ethyl 4-methoxy-3-[4-(methoxycarbonyl)-3-methyl-1,2-oxazol-5-yl]-1,4-dihydroimidazo[5,1-*c*][1,2,4]triazine-8-carboxylate (**14b**): Compound **14b** was synthesized by the same procedure as **14a** from compound **11c** (188 mg, 0.5 mmol), stirred overnight at room temperature in the mixture of methanol (5 mL) and acetic acid (0.5 mL). Yield 158 mg (87%), yellow solid, mp 167–169 °C. ^1^H NMR (400 MHz, CDCl_3_) δ 10.30 (1H, br. s, NH), 7.54 (1H, s, H), 6.96 (1H, s, H), 4.44 (2H, q, *J* = 7.1 Hz, OCH_2_CH_3_), 3.89 (3H, s, OCH_3_), 3.14 (3H, s, OCH_3_), 2.48 (3H, s, CH_3_), 1.42 (3H, t, *J* = 7.1 Hz, OCH_2_CH_3_). ^13^C NMR (101 MHz, CDCl_3_) δ 166.5, 163.4, 162.1, 160.1, 133.9, 129.2, 125.9, 111.3, 110.6, 75.2, 61.0, 52.9, 52.3, 14.5, 11.8. HRMS (ESI^+^), *m*/*z* calcd. for C_15_H_17_N_5_O_6_ [M+H]^+^ 364.1252, found 364.1254. Found, %: C 49.38, H 4.67, N 19.47. C_15_H_17_N_5_O_6_. Calculated, %: C 49.59, H 4.72, N 19.28.

### 3.2. Photophysical Study

UV–Vis absorption spectra were recorded on a Shimadzu UV-1800 spectrophotometer (Kyoto, Japan). Fluorescence of the sample solutions was measured using a Hitachi F-7000 spectrophotometer (Tokyo, Japan). The absorption and emission spectra were recorded in toluene, dioxane, CH_2_Cl_2_, CHCl_3_, THF, EtOH, EtOAc, acetone, MeCN, DMF, and DMSO using 10.00 mm quartz cells. The excitation wavelength was at the absorption maxima. Atmospheric oxygen contained in solutions was not removed. Concentration of the compounds in the solution was 5.0 × 10**^−^**^5^ M and 5.0 × 10**^−^**^6^ M for absorption and fluorescence measurements, respectively. The relative fluorescence quantum yields (QY) were determined using quinine sulfate (*c* = 5 × 10**^−^**^5^ M) in 0.1 M H_2_SO_4_ as a standard (Φ_F_ = 0.546).

AIE/AIEE phenomena study: A stock solution of the investigated compound in THF (or DMSO) (5 × 10**^−^**^4^ M) was prepared and an aliquot (1.0 mL) of this solution transferred to a 25 mL volumetric flask. After addition of a calculated volume of pure solvent, water was added at once to prepare mixtures with water content in the range of 0–90 vol% and final concentration 5 × 10**^−^**^5^ M. The UV–Vis and fluorescence measurements of the resultant solutions were then performed immediately. The absolute quantum yield for the solid-state and time-resolution study was recorded on a Horiba FlouroMax 4 Spectrofluorometer (Kyoto, Japan) with a Quanta-φ integrating sphere using the FluorEssence 3.5 Software (Kyoto, Japan).

## 4. Conclusions

In summary, we synthesized new assemblies of 1,4-dihydroazolotriazines and isoxazoles and characterized them using ^1^H and ^13^C NMR, HRESMS, and elemental analysis. The DATs obtained demonstrated the stability of their bicyclic systems, although C4–NMe_2_ is easily substituted by the MeO and OH groups. The DATs demonstrate emissions in organic solvents ranging from 433 to 487 nm, with QYs of 6.1–33.3% and a high Stokes shift (up to150 nm). Studies of the XRD data of the two DAT patterns demonstrate their tendency to form supramolecular architectures due to the ability to form a large amount of intra- and intermolecular NCIs. New non-aromatic azolotriazines demonstrated fluorescence in powder with a high quantum yield up to 95%.

DATs exhibited different sensitivities to the microenvironment depending on the structure of the azole ring involved in the central bicyclic scaffold. Imidazole derivatives **11** showed positive solvato(fluoro)chromism, with the best QYs in non-polar toluene (38%). The pyrazole derivative **10** only exhibited fluorescence in protic solvents and chloroform. Moreover, 1,4-dihydropyrasolotriazine had an excellent AIE effect in a DMSO–H_2_O and THF–H_2_O binary mixture with the increasing of QYs up to 16 and 13%, respectively. Analysis of the DAT behavior in different solvents with Lippert–Mataga and Dimroth–Reichardt plots established the strong influence of the nature of C4 substituents. Thus, the DATs’ photophysical properties are very sensitive to structural fragments and can be tuned by various types of modification. Structural diversity can be provided by using different starting heterocyclic diazo compounds and enamines and employing the nucleophilic substitution of the NMe_2_ group by numerous natural and bioactive compounds.

## Figures and Tables

**Table 2:** The fluorescence lifetime (<τ>_f_) and radiative (*k*_r_) and non-radiative (*k*_nr_) decay rate constants of DATs **10a**, **11a**–**c**, **13a**, and **14a**,**b** solutions in CHCl_3_.

Entry	Compd.	<τ>_f_ (ns)	*k*_r_ × 10^−9^ (s^−1^)	*k*_nr_ × 10^−9^ (s^−1^)	*k*_nr_/*k*_r_
1	**10**	1.577	0.039	0.595	15.3
2	**11a**	0.369	0.515	2.195	4.3
3	**11b**	2.220	0.055	0.426	7.7
4	**11c**	2.553	0.066	0.326	4.9
5	**13a**	3.318	0.088	0.213	2.4
6	**14a**	2.307	0.144	0.289	2.0
7	**14b**	2.781	0.113	0.247	2.2

**Table 3:** Photophysical characteristics of DATs **11a**,**c**, **13a**, and **14a**,**b** in a solid state.

Entry	Compd.	λ*_ex_*, nm	λ*_em_*, nm	QY *^a^*, %
1	**11a**	429	472	34.5
2	**11c**	420	504	19.6
3	**13a**	410	484	62.1
4	**14a**	420	476	98.7
5	**14b**	415	494	9.8

*^a^*—Absolute QY measured by integrated sphere.

**Table 4:** Photophysical characteristics of DATs **10**, **11a**,**c**, **13a**, and **14a**,**b** solutions in DMSO and DMSO–H_2_O mixture (*v*/*v*, 1/9) (*c* = 1 × 10^−5^ M).

Entry	Compd	Solvent	λ_max_, nm	ε, M^−1^·cm^−1^	λ_em_, nm	QY *^a^*, %	Stokes Shift, nm/cm^−1^
1	**10**	DMSO	344	10,400	–	–	–
2		DMSO–H_2_O	340	12,300	459	9	119/7625
3	**11a**	DMSO	364	22,400	492	3	128/7147
4		DMSO–H_2_O	358	21,700	503	4	145/8052
5	**11c**	DMSO	356	15,200	497	5	141/7969
6		DMSO–H_2_O	352	14,800	503	6	151/8528
7	**13a**	DMSO	361	16,300	499	19	138/7661
8		DMSO–H_2_O	355	18,700	505	4	150/8367
9	**14a**	DMSO	366	23,000	496	24	130/7161
10		DMSO–H_2_O	359	24,000	501	5	142/7895
11	**14b**	DMSO	362	17,100	499	19	137/7584
12		DMSO–H_2_O	358	18,100	502	5	144/8013

*^a^*—quantum yield (QY) is measured relative to quinine sulfate (λ_exc_ = 366 nm; Φ_F_ = 0.53) and calculated using established equations.

## Data Availability

The data presented in this study are available in the Supplementary Materials.

## Supplementary Materials

The following supporting information can be downloaded at: https://www.mdpi.com/article/10.3390/molecules28073192/s1. ^1^H and ^13^C nuclear magnetic resonance (NMR) spectroscopy; UV–Vis and fluorescent spectra and photophysical characteristics. X-ray data of compounds **11b** and **13b**. These experiments were accomplished on the automated X-ray diffractometer «Xcalibur 3» with CCD detector on standard procedure (MoKα-irradiation, graphite monochromator, ω-scans with 1o step at T = 295(2) K). Empirical absorption correction was applied. The solution and refinement of the structures were accomplished with using Olex2 program package [64]. The structure was solved with the Superflip [65] structure solution program using Charge Flipping and refined by ShelXL by full-matrix least-squared method in the anisotropic approximation for non-hydrogen atoms [66]. The H-atoms at C-H bonds were placed in the calculated positions, the H-atoms at N-H bonds were refined independently in isotropic approximation. CCDC 2225550 for **11b** and CCDC 2238176 for **13b** can be obtained free of charge from the Cambridge Crystallographic Data Centre via link www.ccdc.cam.ac.uk/data_request/cif.

## Sample Availability

Samples of compounds **11**–**14** are available from the authors.
