# Supplementary material for: Isoxazolyl-Derived 1,4-Dihydroazolo[5,1-c][1,2,4]Triazines: Synthesis and Photochemical Properties
Source: Molecules. 2023 Apr 3;28(7):3192. doi: 10.3390/molecules28073192 (PMC10095850; doi:10.3390/molecules28073192)
Supplement: Supplementary file 1 [file molecules-28-03192-s001.zip › molecules-2313541-supplementary.pdf]

Supplementary Material

Isoxazolyl derived 1,4-dihydroazolo[5,1-*c*][1,2,4]triazines: synthesis and photochemical properties

Elena V. Sadchikova[1]*, Nikita E. Safronov[1],

Nikolai A. Beliaev[1], Valentine G. Nenajdenko[2]*, Nataliya P. Belskaya[1]*

[1] Ural Federal University, Ekaterinburg, 620002, Russia
[2] Moscow State University, Moscow, 119992, Russia

Corresponding Authors

Dr. Sadchikova Elena, E-mail: e.v.sadchikova@urfu.ru

Prof. Nenajdenko Valentine, E-mail: nenajdenko@gmail.com

Prof. Belskaya Nataliya, E-mail: n.p.belskaya@urfu.ru

Table of contents

1. ^{1}H, ^{13}C NMR spectra of new compounds S2

2. X-ray structural analysis of compound **11b** and **13b** S15

3. Photophysical study S18

4. Reference S27

1. NMR spectra of compounds 10, 11a–d, 12, 13a,b, 14a,b

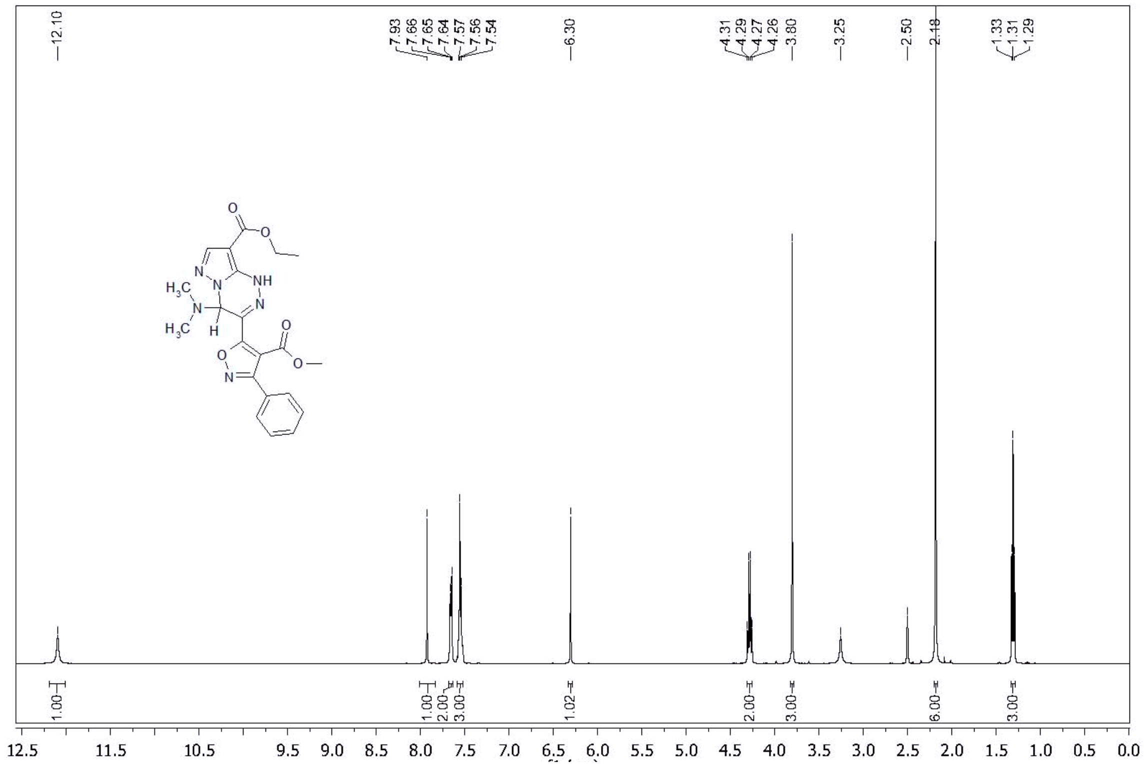

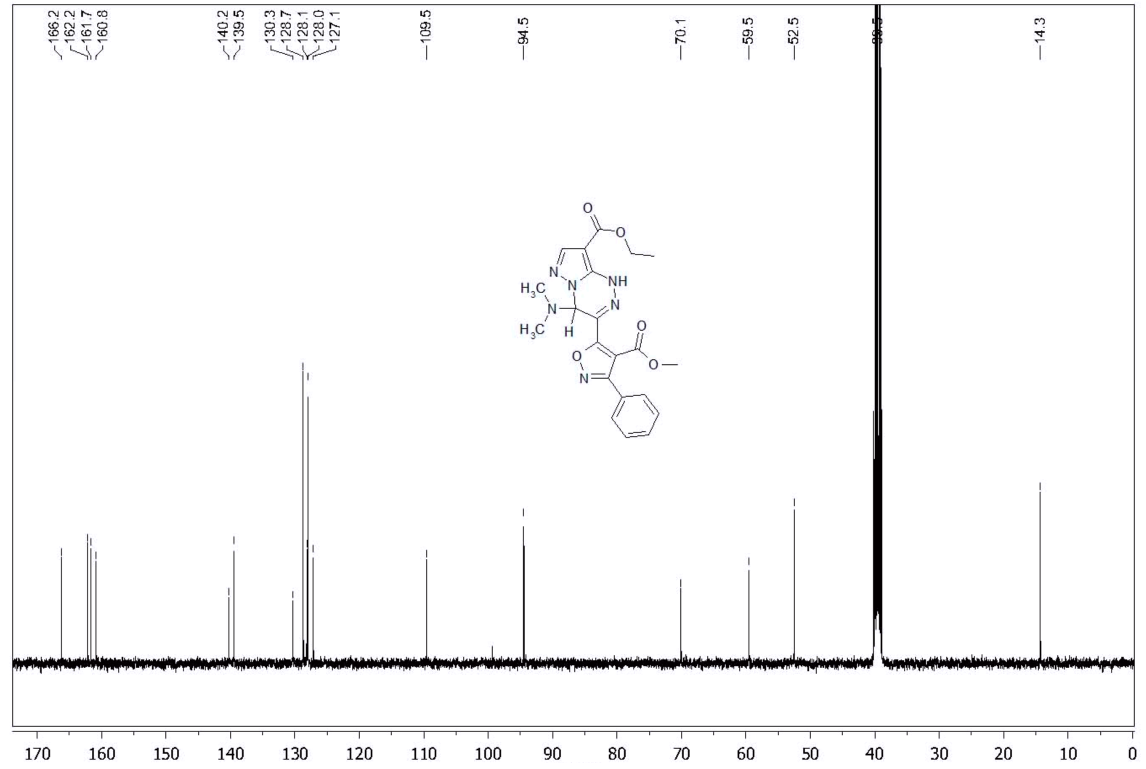

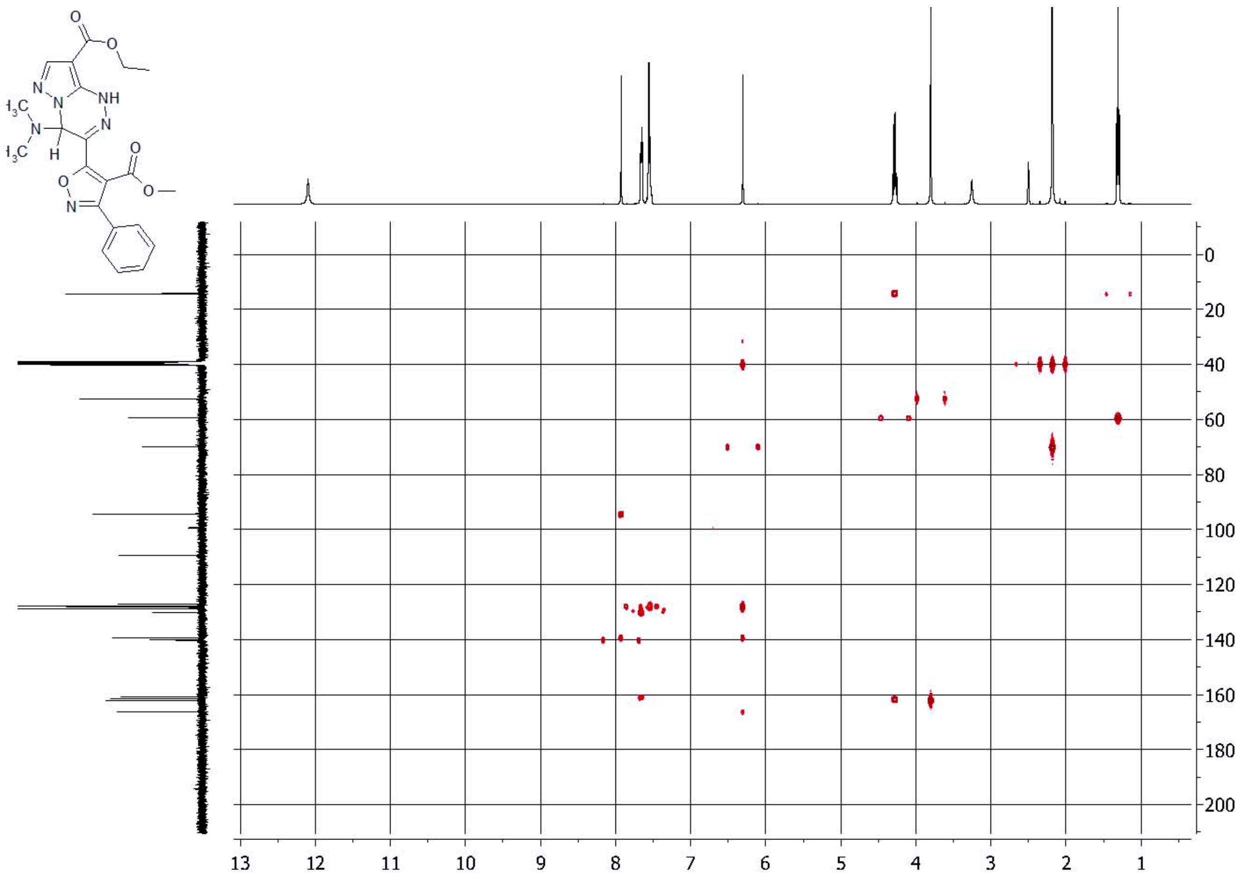

Figure S1. ^{1}H, ^{13}C and HMBC NMR spectra of ethyl 4-(*N*,*N*-dimethylamino)-3-[4-(metoxycarbonyl)-3-phenyl-1,2-oxazol-5-yl]-1,4-dihydropyrazolo[5,1-*c*][1,2,4]triazine-8-carboxylate (**10**)

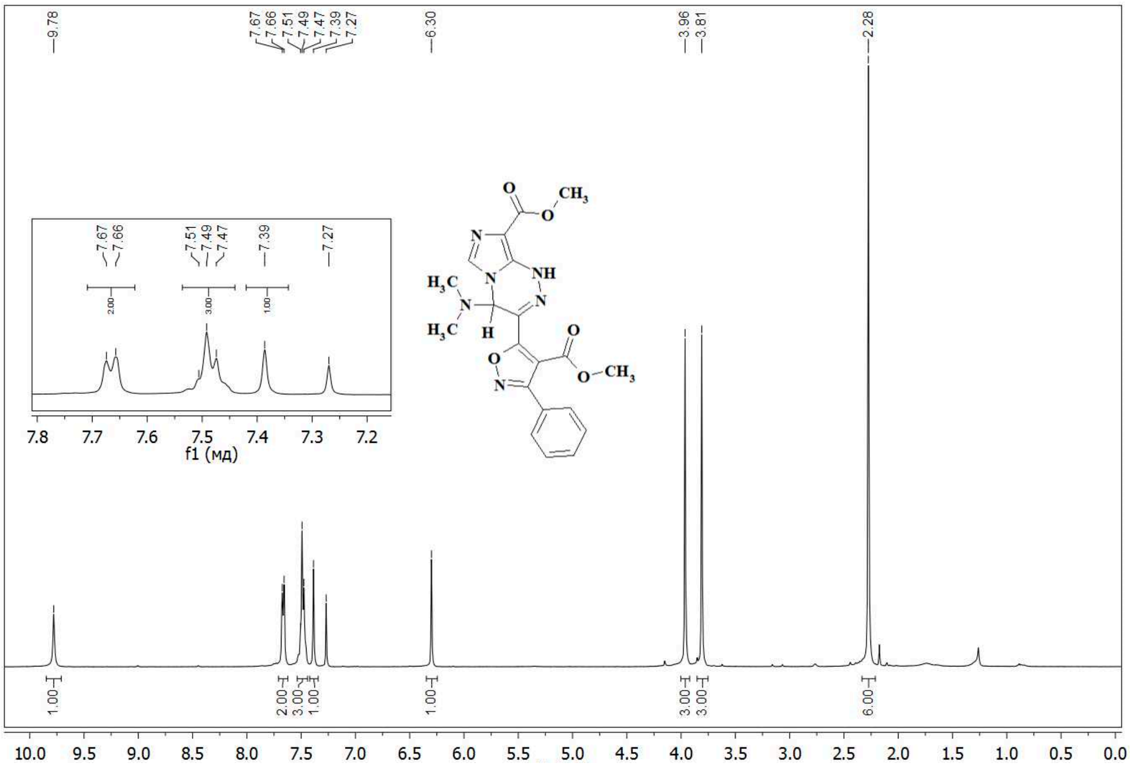

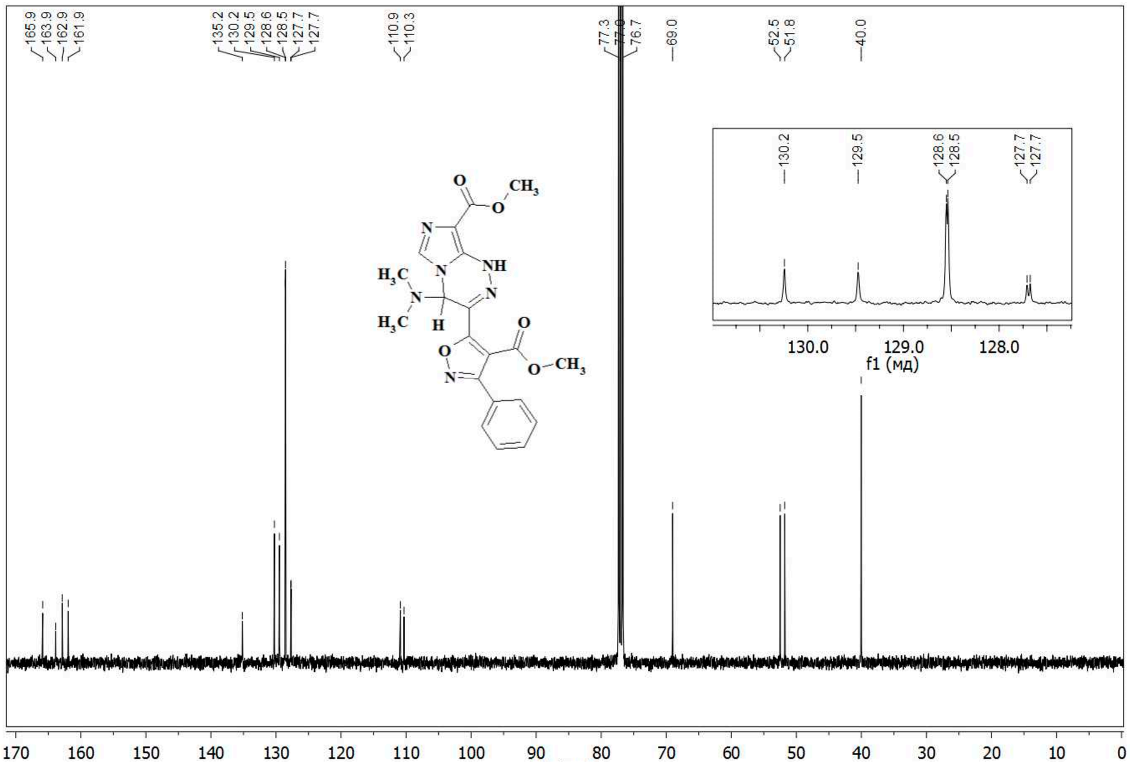

Figure S2.^{1}H and ^{13}C NMR spectrum of methyl 4-(*N*,*N*-dimethylamino)-3-[4-(methoxycarbonyl)-3-phenyl-1,2-oxazol-5-yl]-1,4-dihydroimidazo[5,1-*c*][1,2,4]triazine-8-carboxylate (**11a**)

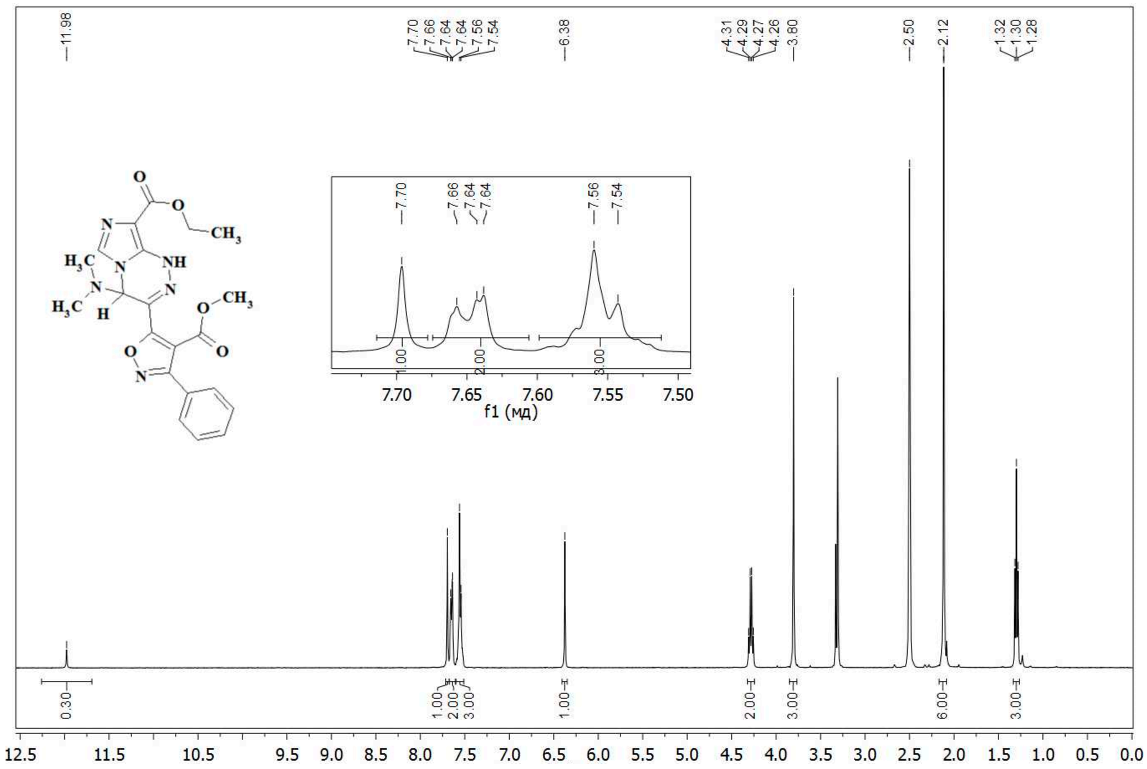

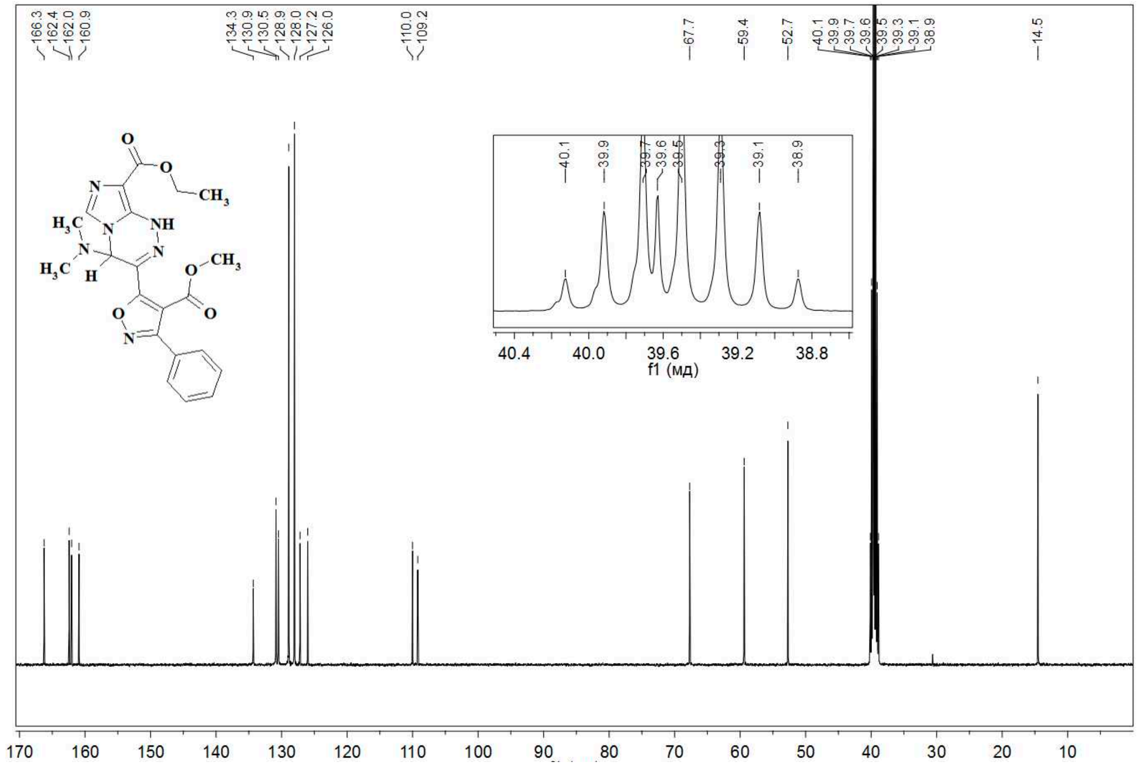

Figure S3. ^{1}H and ^{13}C NMR spectra of ethyl 4-(*N*,*N*-dimethylamino)-3-[4-(methoxycarbonyl)-3-phenyl-1,2-oxazol-5-yl]-1,4-dihydroimidazo[5,1-*c*][1,2,4]triazine-8-carboxylate (**11b**)

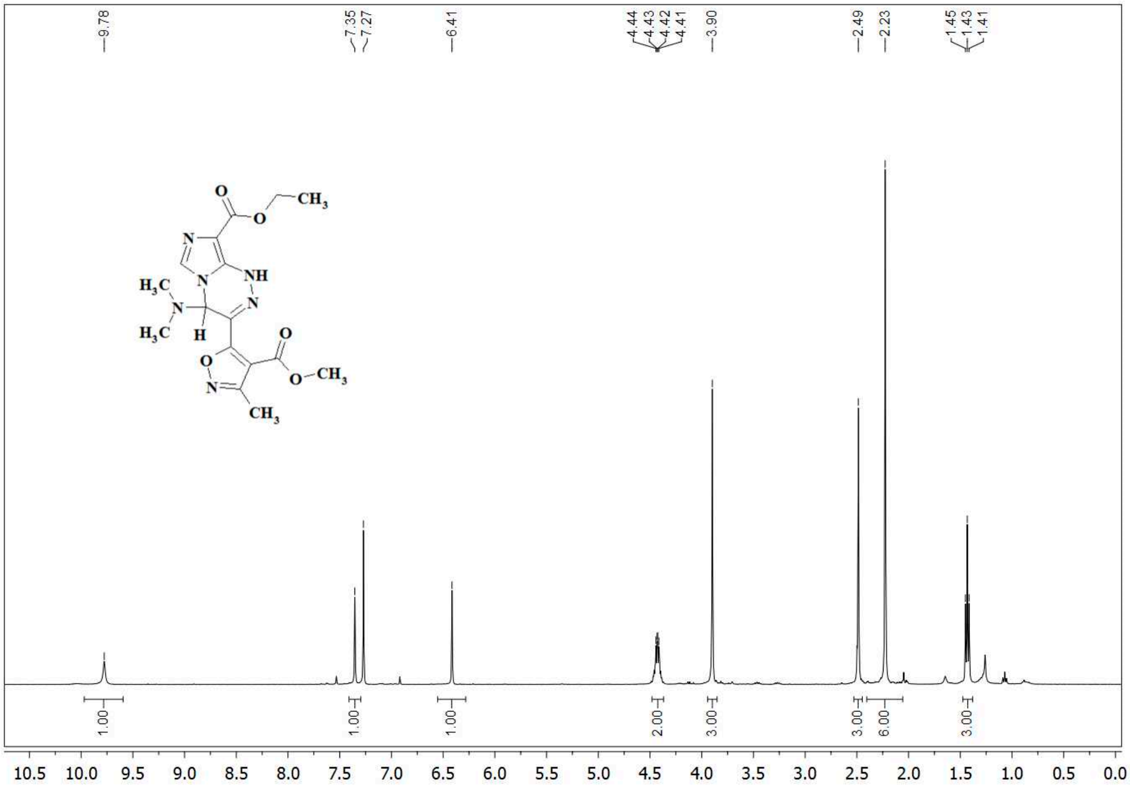

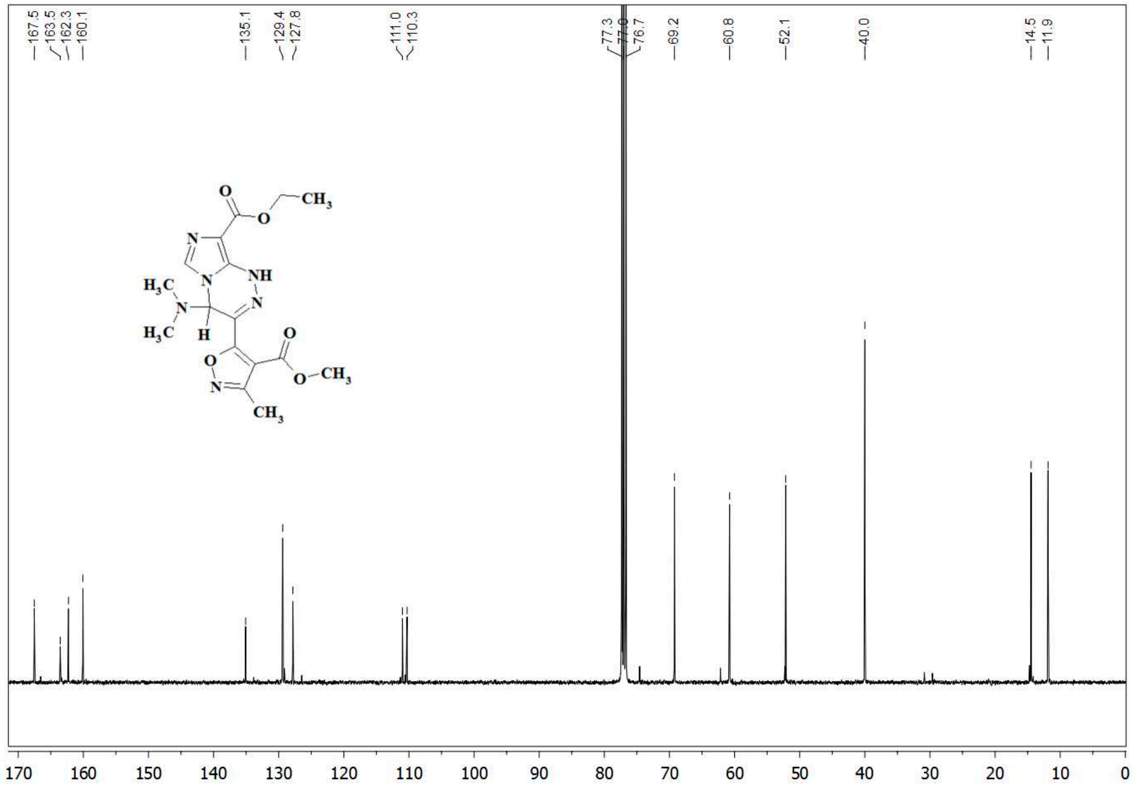

Figure S4. ^{1}H and ^{13}C NMR spectra of ethyl 4-(*N*,*N*-dimethylamino)-3-[4-(methoxycarbonyl)-3-methyl-1,2-oxazol-5-yl]-1,4-dihydroimidazo[5,1-*c*][1,2,4]triazine-8-carboxylate (**11c**)

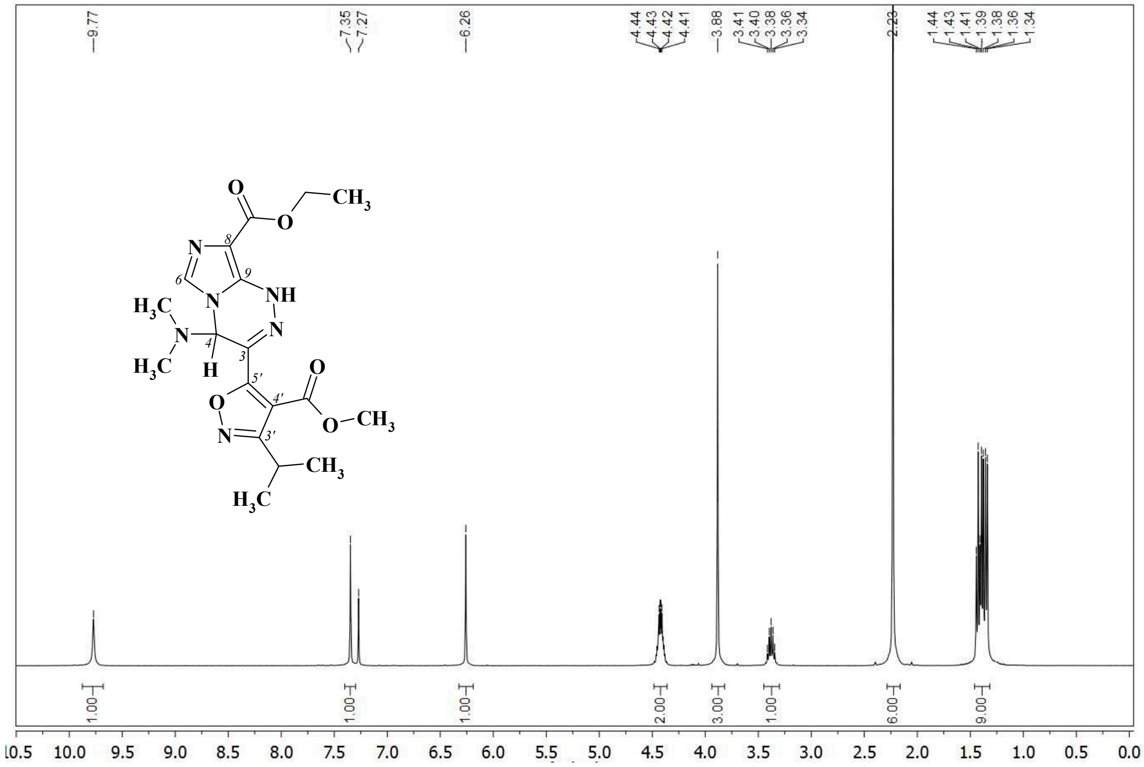

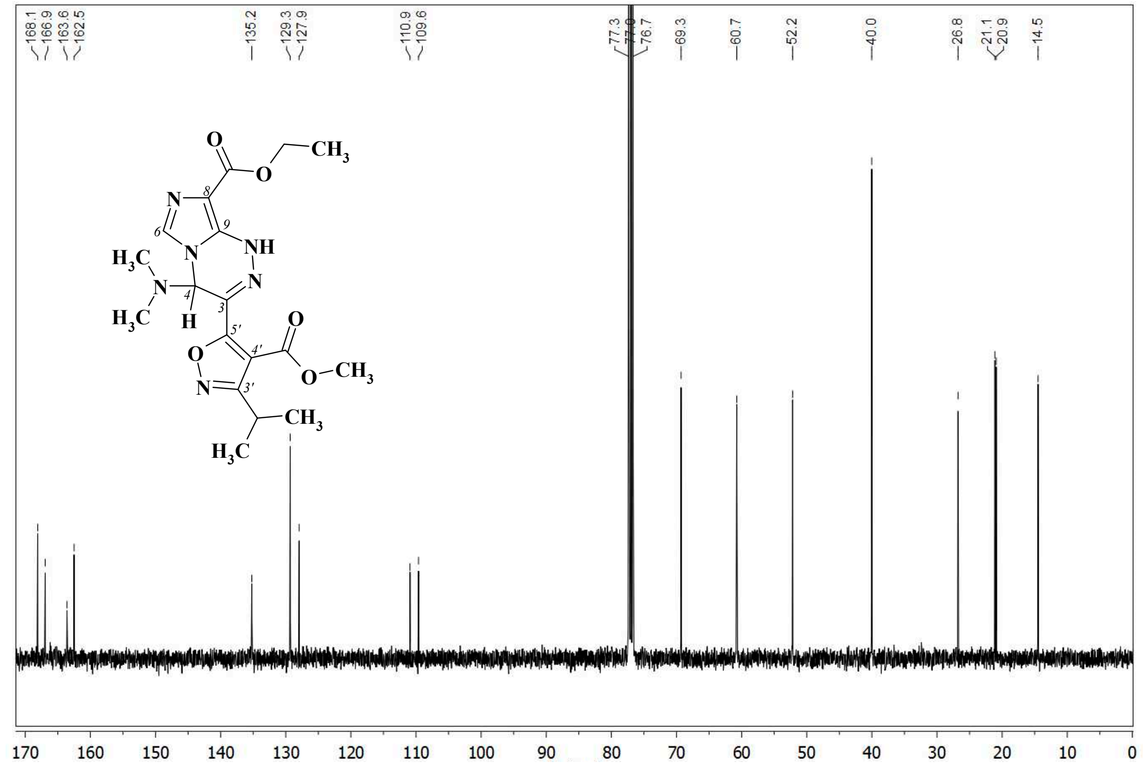

Figure S5. ^{1}H and ^{13}C NMR spectra of ethyl 4-(*N*,*N*-dimethylamino)-3-[3-isopropyl-4-(metoxycarbonyl)-1,2-oxazol-5-yl]-1,4-dihydroimidazo[5,1-*c*][1,2,4]triazine-8-carboxylate (**11d**)

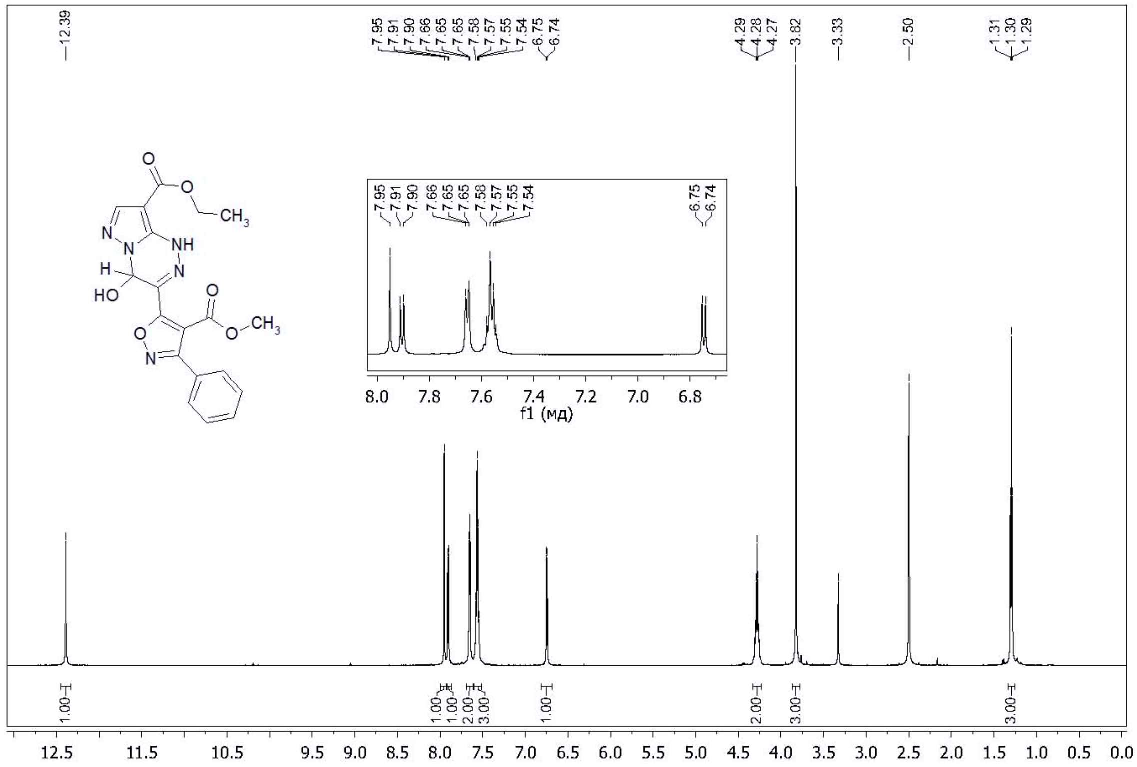

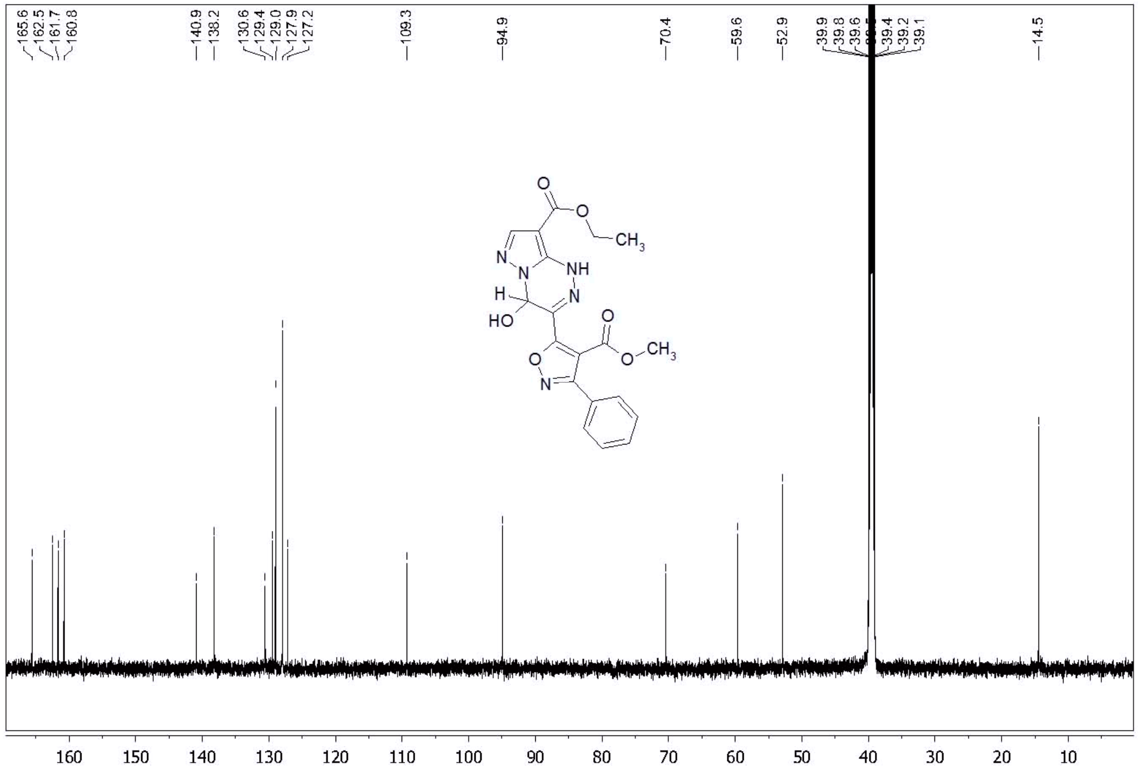

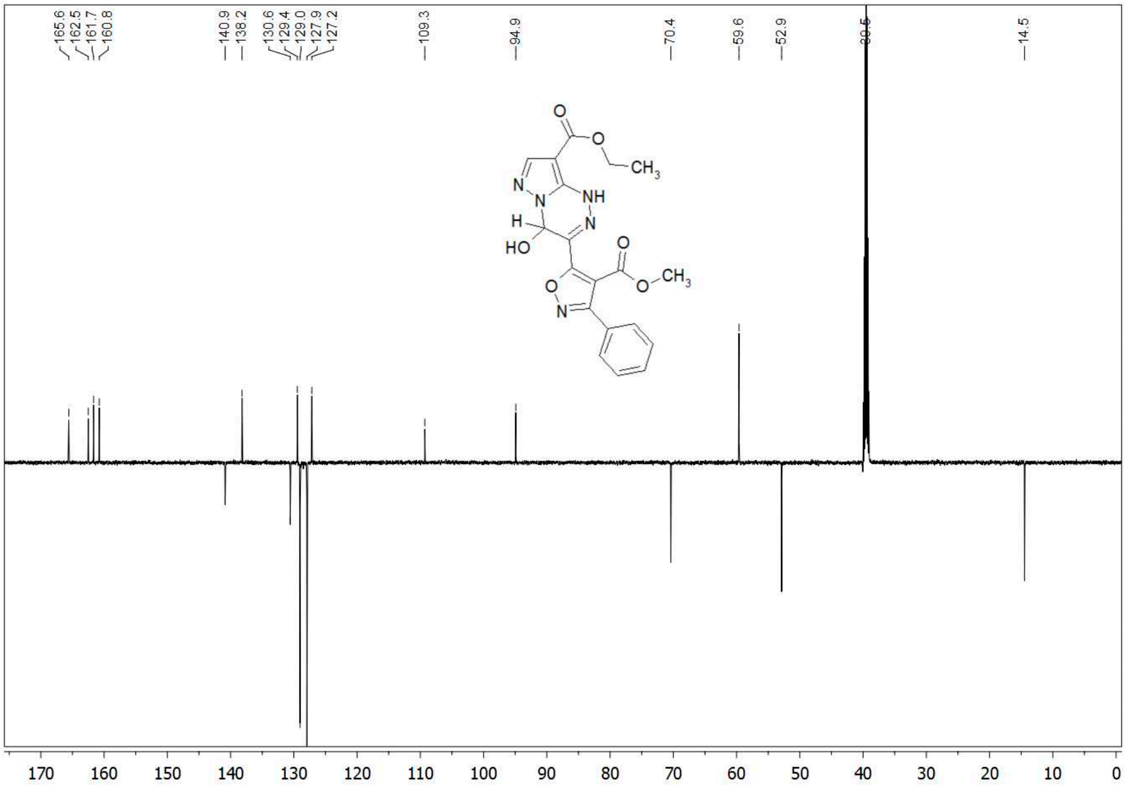

Figure S6. ^{1}H, ^{13}C (BB) and ^{13}C(apt) NMR spectra of ethyl 4-hydroxy-3-[4-(methoxycarbonyl)-3-phenyl-1,2-oxazol-5-yl]-1,4-dihydropyrazolo[5,1-*c*][1,2,4]triazine-8-carboxylate (**12**)

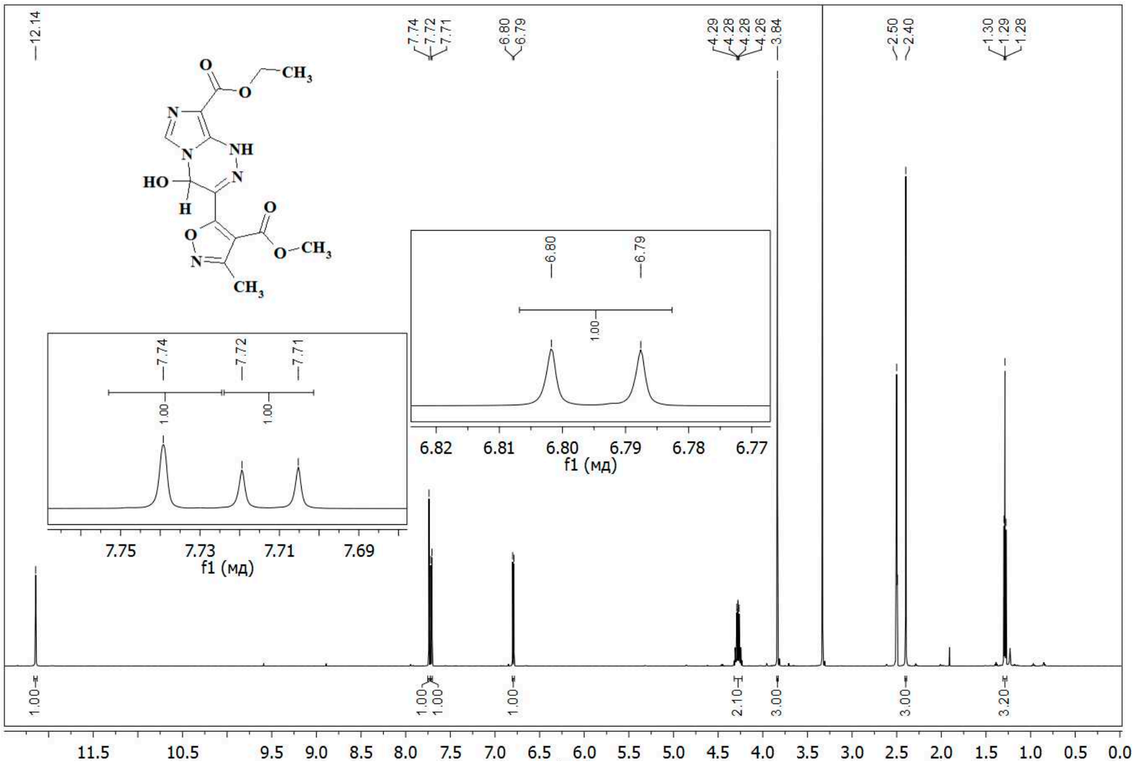

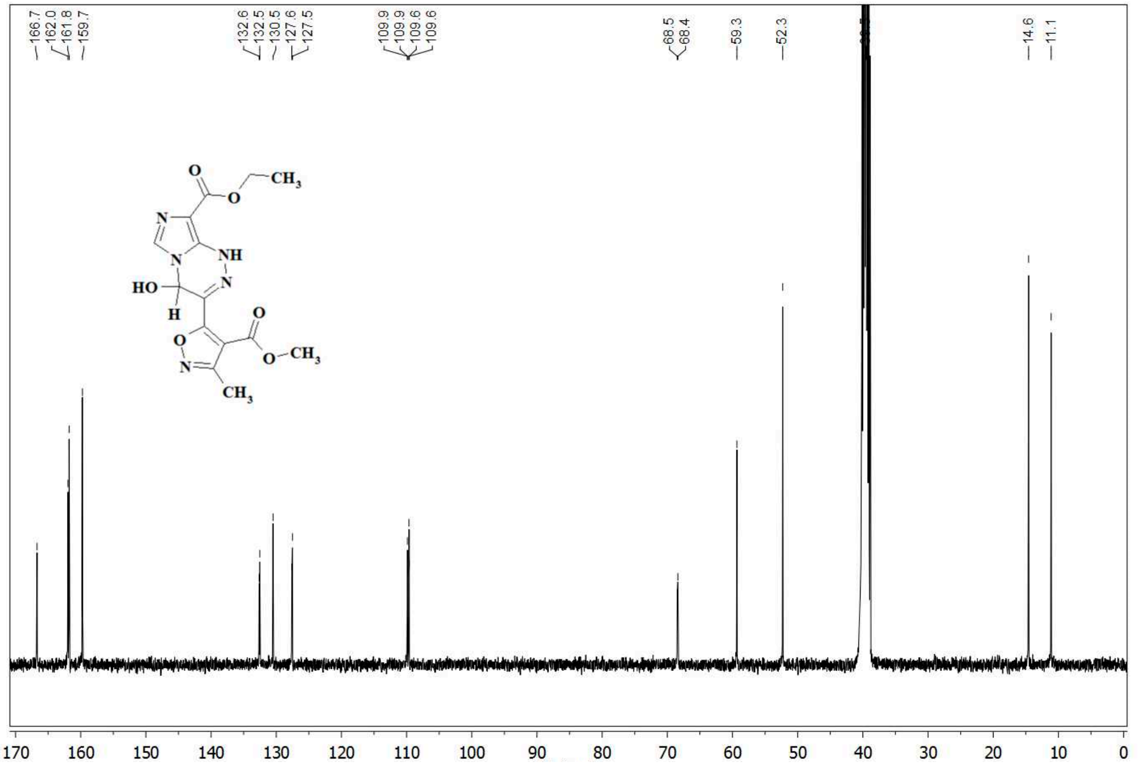

Figure S7. ^{1}H and ^{13}C NMR spectra of ethyl 4-hydroxy-3-[4-(methoxycarbonyl)-3-methyl-1,2-oxazol-5-yl]-1,4-dihydroimidazo[5,1-*c*][1,2,4]triazine-8-carboxylate (**13a**)

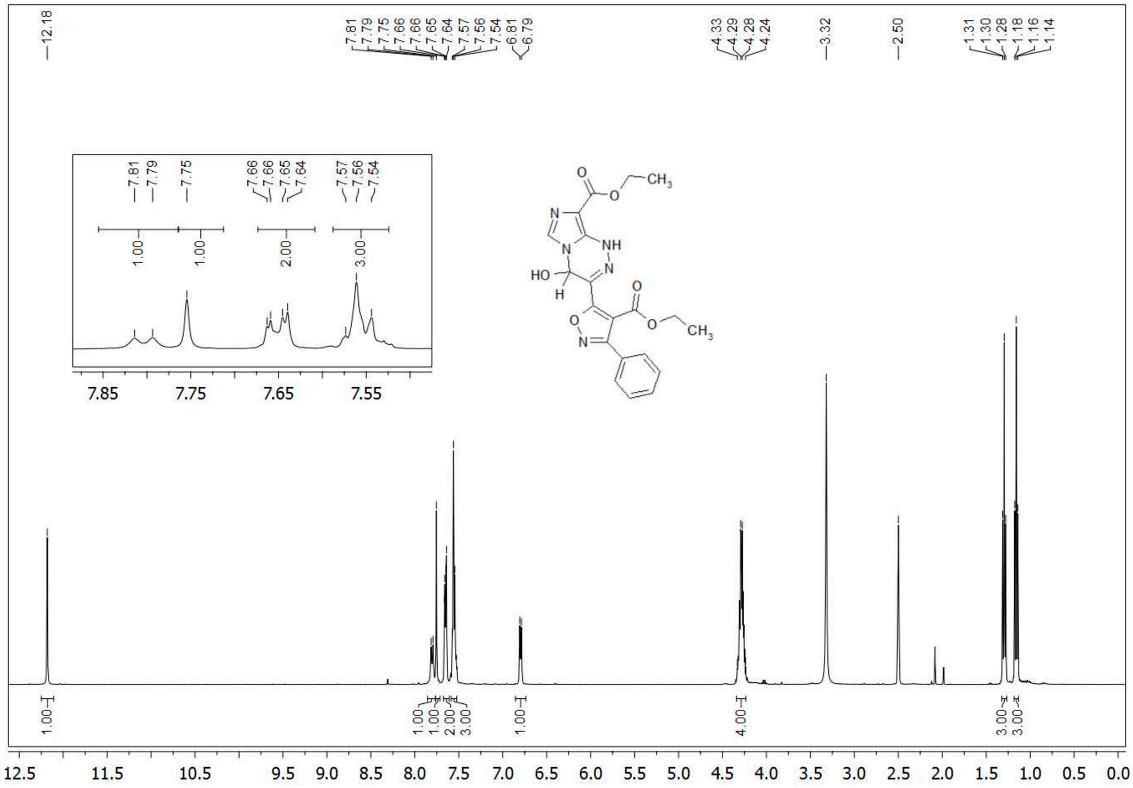
12.18
7.81
7.79
7.75
7.66
7.66
7.65
7.64
7.57
7.56
7.54
6.81
6.79
4.33
4.29
4.28
4.24
3.32
2.50
1.31
1.30
1.28
1.18
1.16
1.14
HO
H
NH
N
O
CH3
12.5 11.5 10.5 9.5 9.0 8.5 8.0 7.5 7.0 6.5 6.0 5.5 5.0 4.5 4.0 3.5 3.0 2.5 2.0 1.5 1.0 0.5 0.0

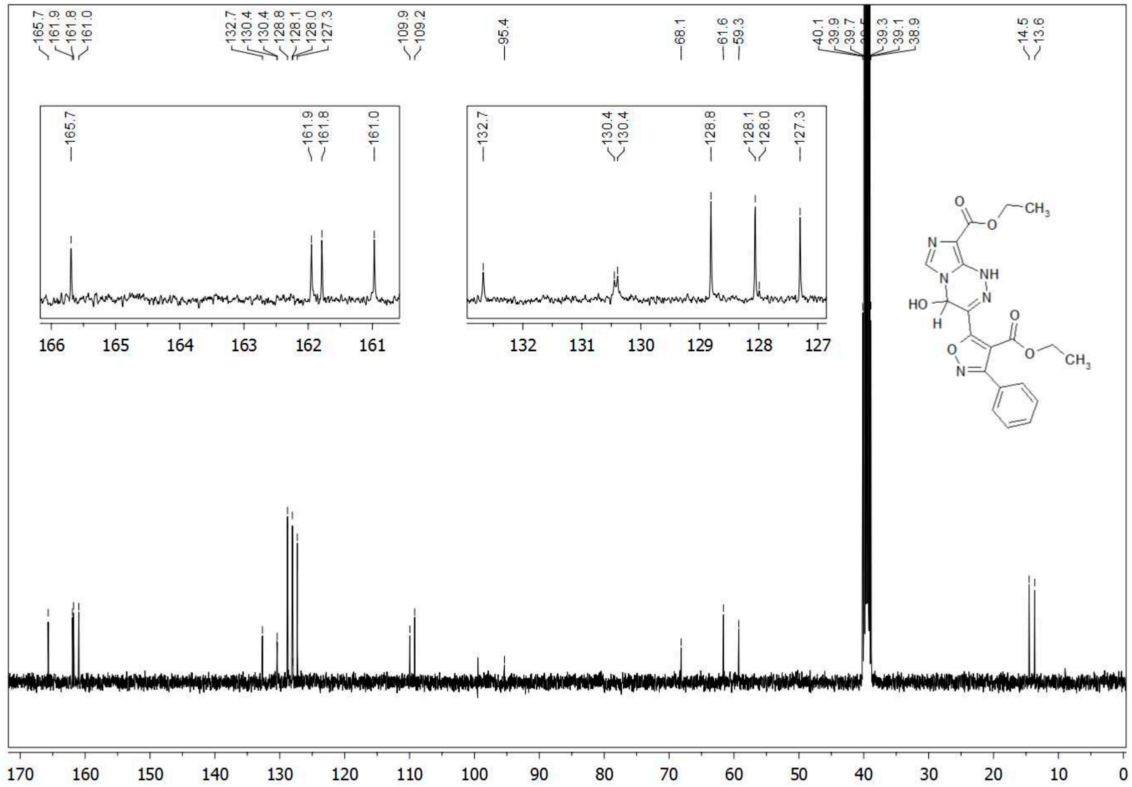
165.7
161.9
161.8
161.0
132.7
130.4
130.4
128.8
128.1
128.0
127.3
109.9
109.2
95.4
68.1
61.6
59.3
40.1
39.9
39.7
39.3
39.1
38.9
14.5
13.6
170 160 150 140 130 120 110 100 90 80 70 60 50 40 30 20 10 0

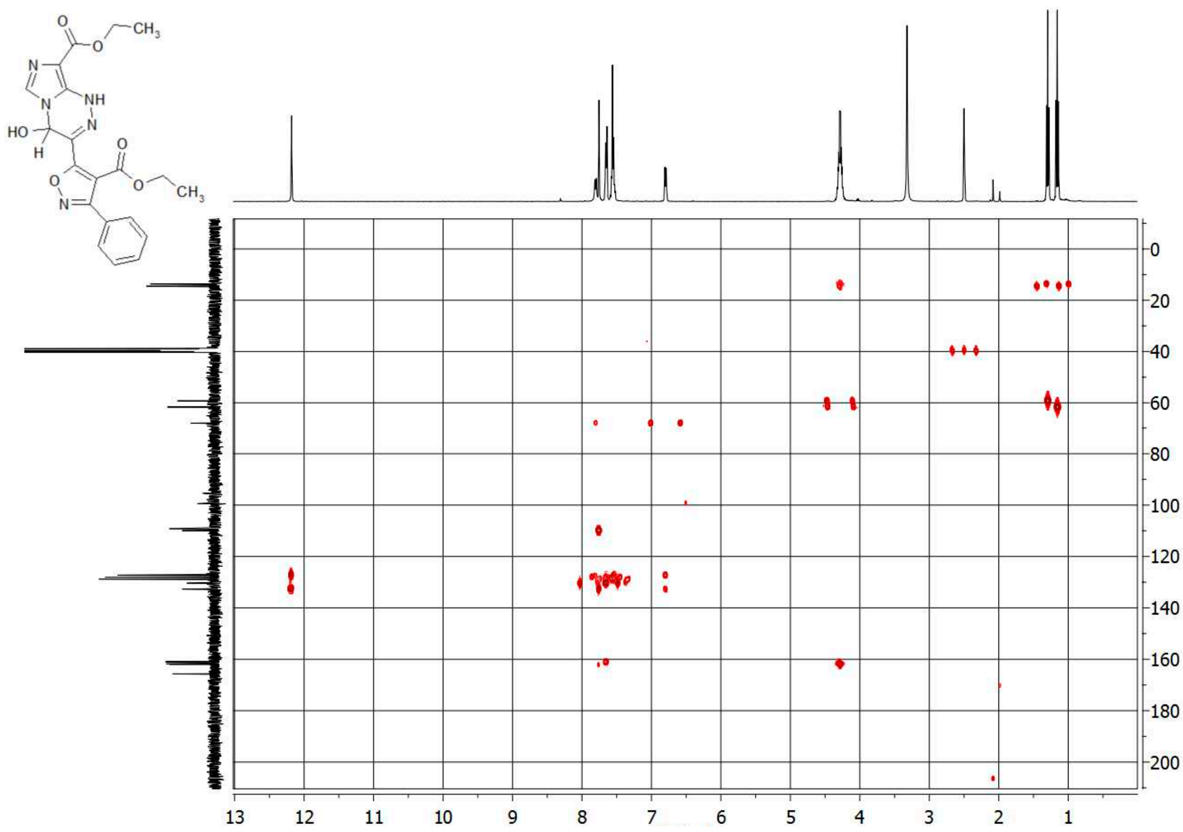

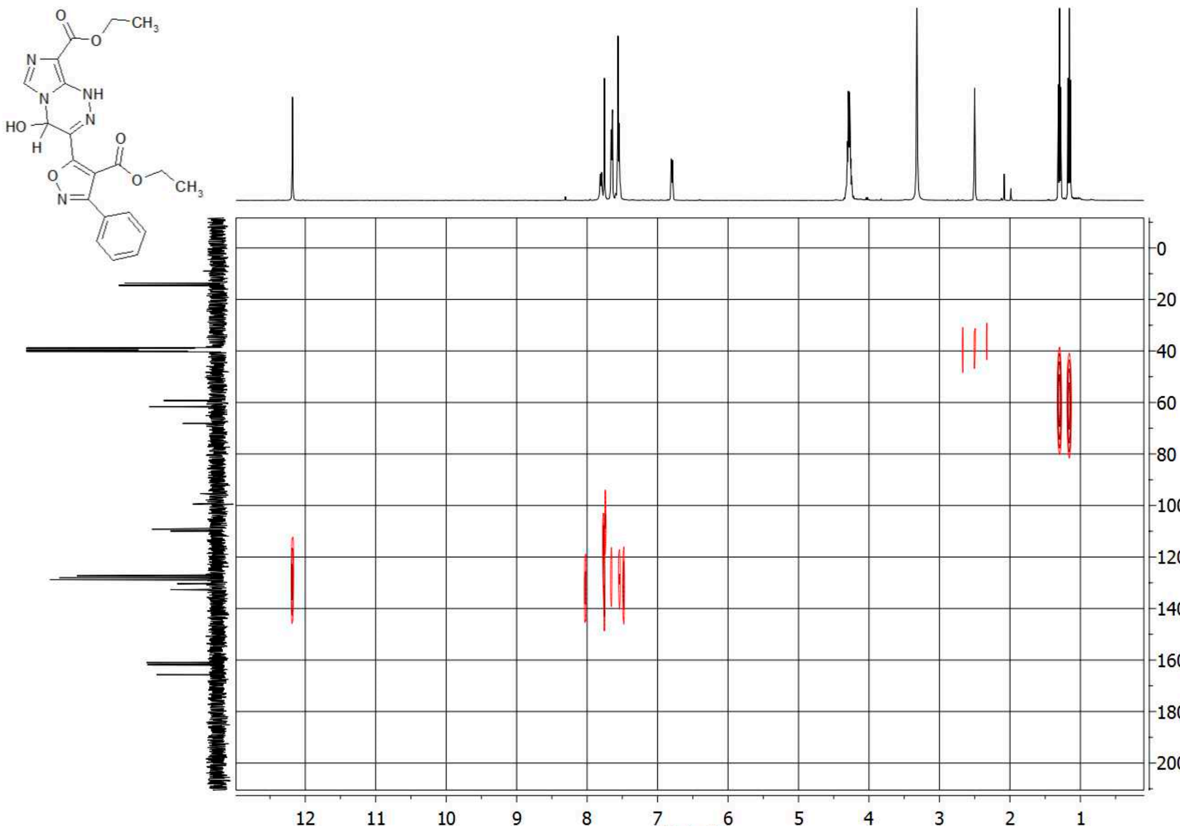

Figure S8. ^{1}H, ^{13}C, HMBC and HSQC spectra of ethyl 3-[4-(ethoxycarbonyl)-3-phenyl-1,2-oxazol-5-yl]-4-hydroxy-1,4-dihydroimidazo[5,1-*c*][1,2,4]triazine-8-carboxylate (**13b**)

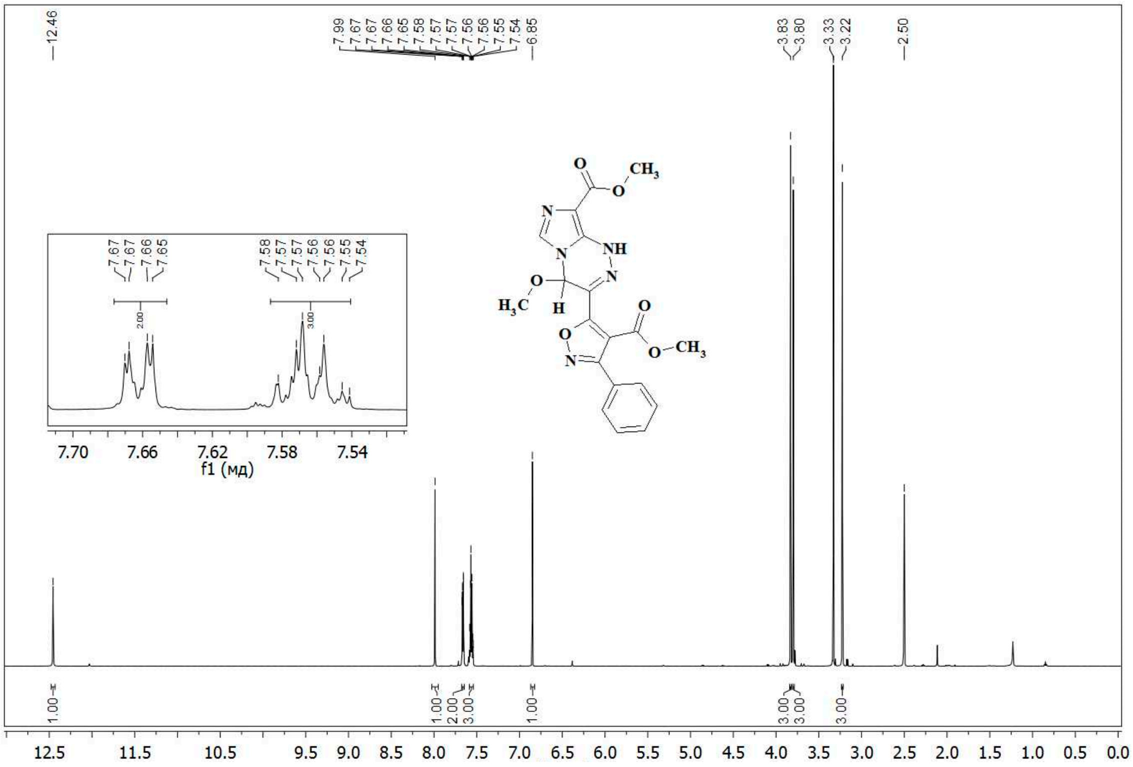

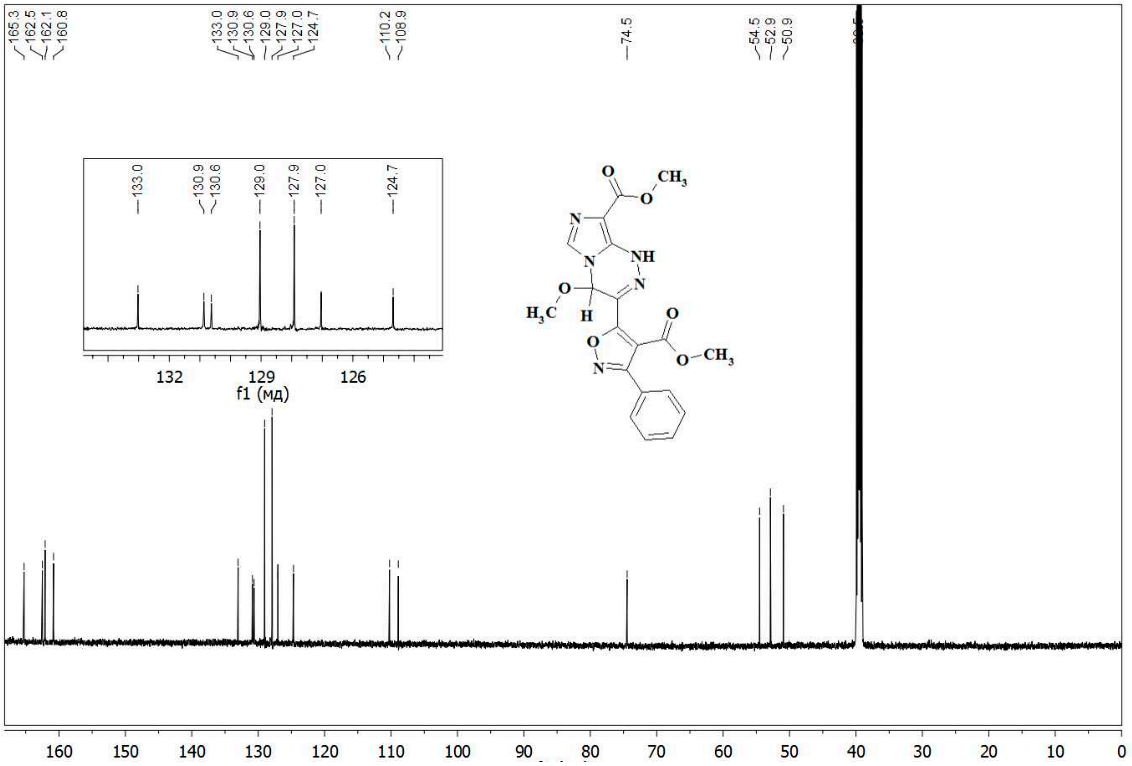

Figure S9. ^{1}H and ^{13}C NMR spectra of methyl 4-methoxy-3-[4-(methoxycarbonyl)-3-phenyl-1,2-oxazol-5-yl]-1,4-dihydroimidazo[5,1-*c*][1,2,4]triazine-8-carboxylate (**14a**)

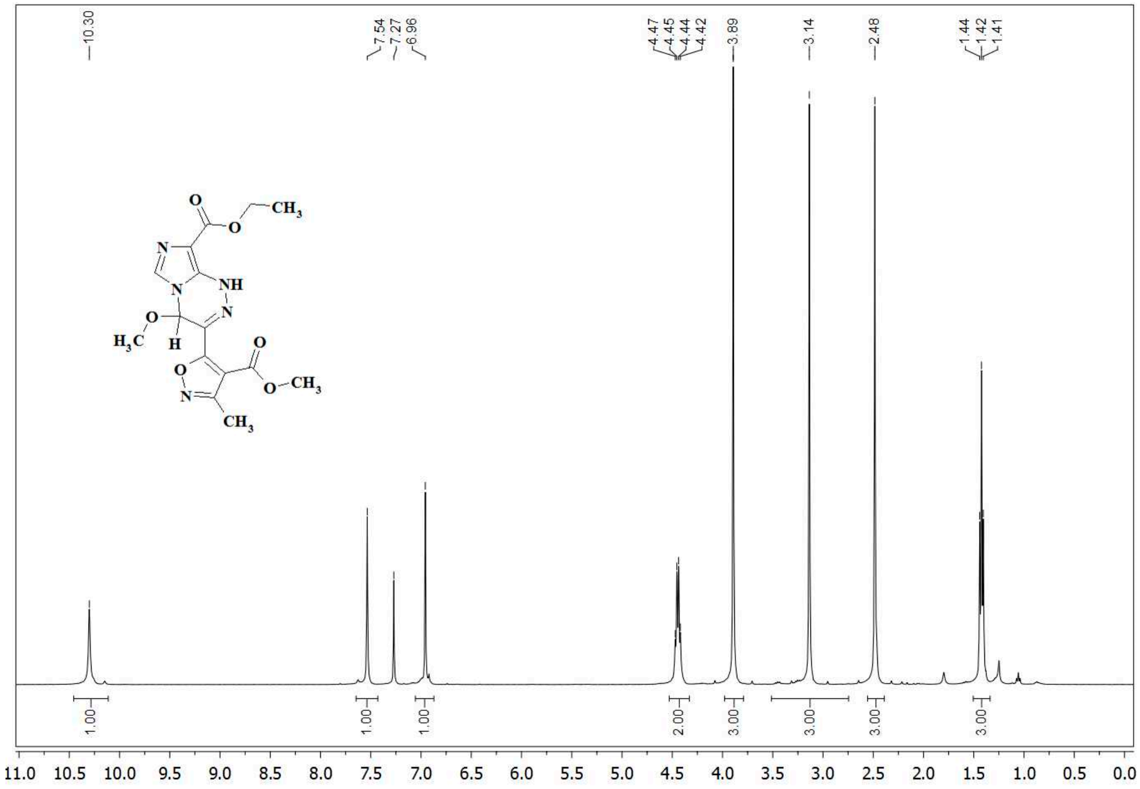

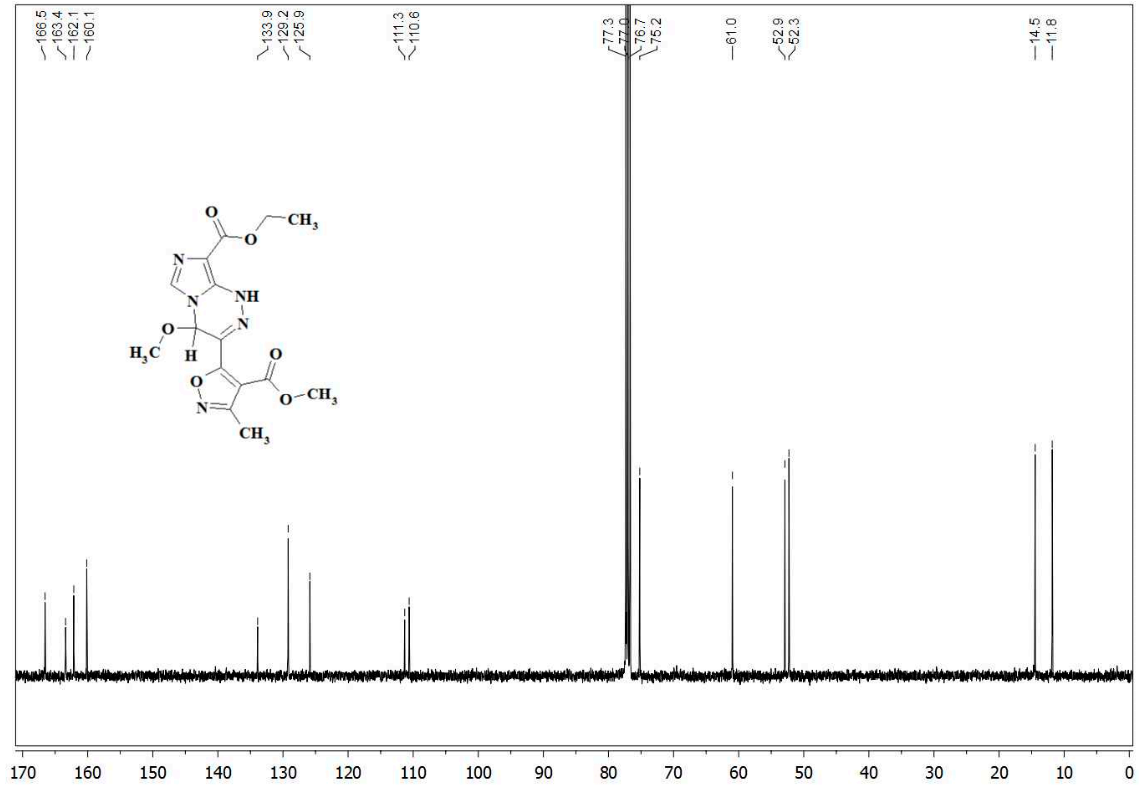

Figure S10. ^{1}H and ^{13}C NMR spectra of ethyl 4-methoxy-3-[4-(methoxycarbonyl)-3-methyl-1,2-oxazol-5-yl]-1,4-dihydroimidazo[5,1-*c*][1,2,4]triazine-8-carboxylate (**14b**)

2. X-ray structural analysis of compounds 11b and 13b

The experiment was accomplished on the automated X-ray diffractometer «Xcalibur 3» with CCD detector on standard procedure (MoK$_\alpha$-irradiation, graphite monochromator, ω-scans with 1° step at T= 295(2) K). Empirical absorption correction was applied. The solution and refinement of the structures were accomplished with using Olex2 program package [1]. The structure was solved with the Superflip [2] structure solution program using Charge Flipping and refined by ShelXL by full-matrix least-squared method in the anisotropic approximation for non-hydrogen atoms [3]. The H-atoms at C–H bonds were placed in the calculated positions, the H-atoms at N–H bonds were refined independently in isotropic approximation.

Crystal Data for $C_{14}H_{17}N_5O_4$ (M = 319.33 g/mol) **11b**: monoclinic, space group $P2_1/c$, a = 10.0537(8) Å, b = 4.8787(4) Å, c = 31.6595(19) Å, β = 98.525(7)°, V = 1535.72(19) Å^3, Z = 4, μ(MoK$_\alpha$) = 0.104 mm^{-1}, 18809 reflections measured (4.52° ≤ 2Θ ≤ 52.74°), 3125 unique (R_{int} = 0.0626) which were used in all calculations. The final R_1 = 0.0471, wR_2 = 0.0982 (I > 2σ(I)) and R_1 = 0.1083, wR_2 = 0.1210 (all data). Largest diff. peak/hole 0.16/-0.15 ēÅ^{-3}.

The XRD data were registered in the Cambridge Structural database (CCDC 2184579) can be obtained free of charge *via* https://www.ccdc.cam.ac.uk.

Crystal Data for $C_{14}H_{17}N_5O_4$ (M = 319.33 g/mol) (**13b**): monoclinic, space group $P2_1/c$, a = 10.0537(8) Å, b = 4.8787(4) Å, c = 31.6595(19) Å, β = 98.525(7)°, V = 1535.72(19) Å^3, Z = 4, μ(MoK$_\alpha$) = 0.104 mm^{-1}, 18809 reflections measured (4.52° ≤ 2Θ ≤ 52.74°), 3125 unique (R_{int} = 0.0626) which were used in all calculations. The final R_1 = 0.0471, wR_2 = 0.0982 (I > 2σ(I)) and R_1 = 0.1083, wR_2 = 0.1210 (all data). Largest diff. peak/hole 0.16/-0.15 ēÅ^{-3}.

The XRD data were registered in the Cambridge Structural database (CCDC 2184579) can be obtained free of charge *via* https://www.ccdc.cam.ac.uk.

CCDC 2225550 for **11b** and CCDC 2238176 for **13b** can be obtained free of charge from the Cambridge Crystallographic Data Centre *via* link www.ccdc.cam.ac.uk/data_request/cif.

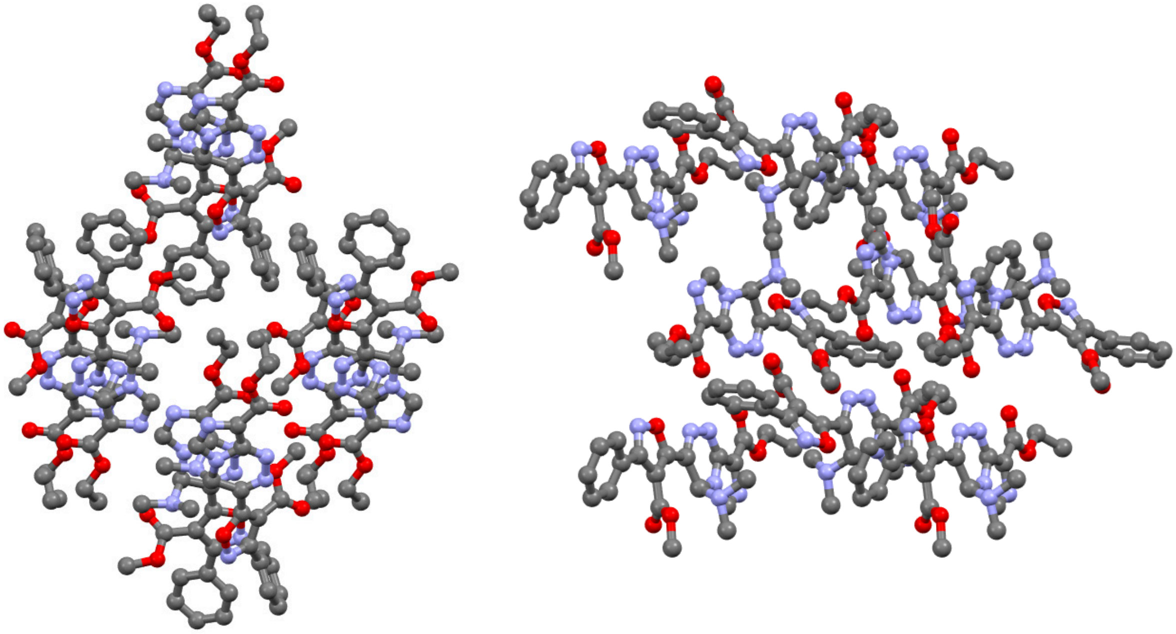

a *b*

Figure S1. The molecular structure of DAT **11b** according to X-ray diffraction data: (*b*) Partial parking diagram along with the crystallographic axis: axis b, and axis c. Hydrogens atoms omitted for clarity.

Table S1. Selected bond lengths and torsion angles in molecule DAT **11b**.

	Bond length, Å					Torsion angles, °		
	C4-C23	C8-C12	C9-N4	C13-C21	C14-C15	N7C8C12C13	C13C14C15C20	N6C5N1C2
Mol 0	1.439	1.465	1.446	1.469	1.486	-34.7(1)	154.5(5)	179.5(8)
Mol A	1.444	1.475	1.438	1.476	1.475	177.7(1)	27.7(5)	179.8(4)
Δ	0.005	0.010	0.008	0.007	0.011	57.0(0)	126.8	0.3(0)

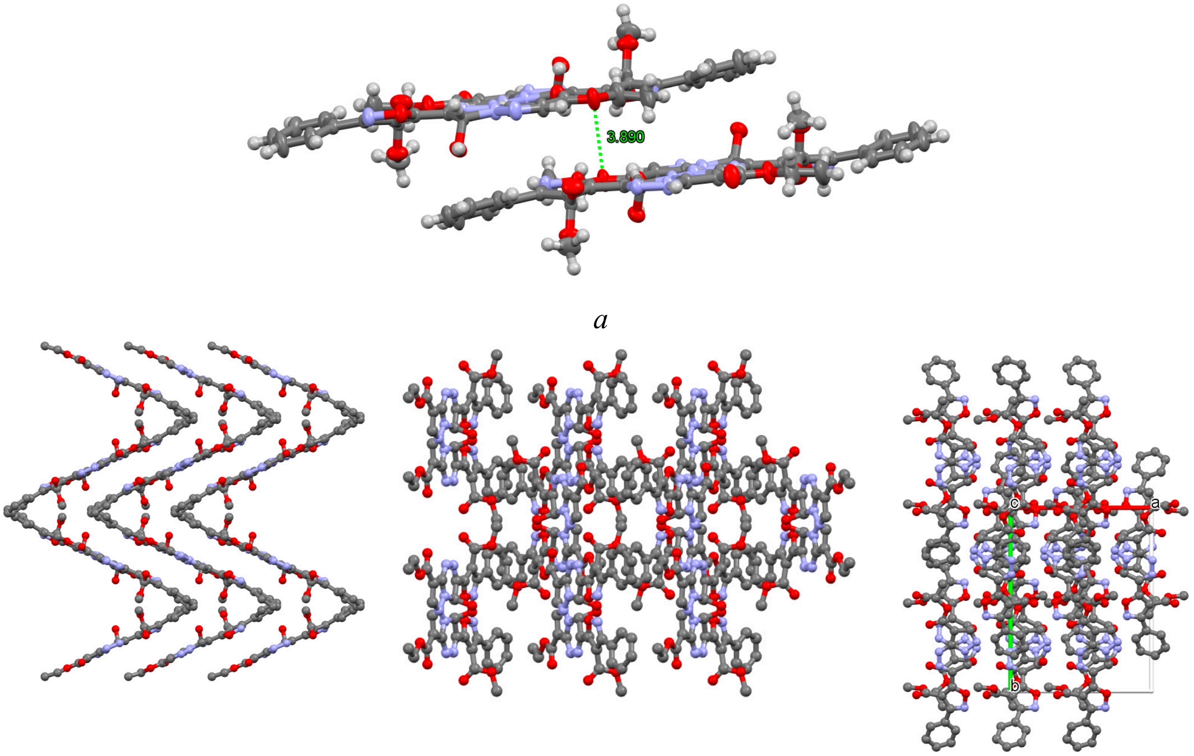

a

b *c* *d*

Figure S2. (*a*) Arrangement of molecules in the two parallel sheets; (*b, c, d*) Partial parking diagram along the crystallographic axis a (*b*), axis b (*c*), and axis c (*d*). Hydrogens atoms omitted for clarity.

Table S2. Selected bond lengths and torsion angles in molecule DAT **11b**.

Bond length, Å					Torsion angle, °		
C1-C2	C6-C7	C9-C10	C8-C16	C5-O2	N4C6C7C8	C8C9C10C15	N3C4N1N2
1.434	1.453	1.477	1.479	1.399	14.59	-24.97	-179.31

3. Photophysical study

UV–Vis absorption spectra were recorded on a Shimadzu UV-1800 spectrophotometer (Kyoto, Japan). Fluorescence of the sample solutions was measured using a Hitachi F-7000 spectrophotometer (Tokyo, Japan). The absorption and emission spectra were recorded in Toluene, Dioxane, CH_2Cl_2, $CHCl_3$, THF, EtOH, EtOAc, Acetone, MeCN, DMF, DMSO using 10.00 mm quartz cells. The excitation wavelength was at the absorption maxima. Atmospheric oxygen contained in solutions was not removed. Concentration of the compounds in the solution was 5.0×10^{-5} M and 5.0×10^{-6} M for absorption and fluorescence measurements, respectively. The relative fluorescence quantum yields (Φ_F) were determined using quinine sulfate (5×10^{-5} M) in 0.1 M H_2SO_4 as a standard ($\Phi_F = 0.546$).

AIE/AIEE phenomena study: A stock solution of investigated compound in THF (or DMSO) ($c = 5 \times 10^{-4}$ M) was prepared and an aliquot (1.0 ml) of this solution transferred to a 25 mL volumetric flask. After addition of a calculated volume of pure solvent, water was added at once to prepare mixtures with water contents in the range of 0–90 vol % and final concentration 5×10^{-5} M. The UV–Vis and fluorescence measurements of the resultant solutions were then performed immediately. The absolute quantum yield for the solid-state and time-resolution study was recorded on Horiba FlouroMax 4 Spectrofluorometer (Kyoto, Japan) with a Quanta-φ integrating sphere using FluorEssence 3.5 Software.

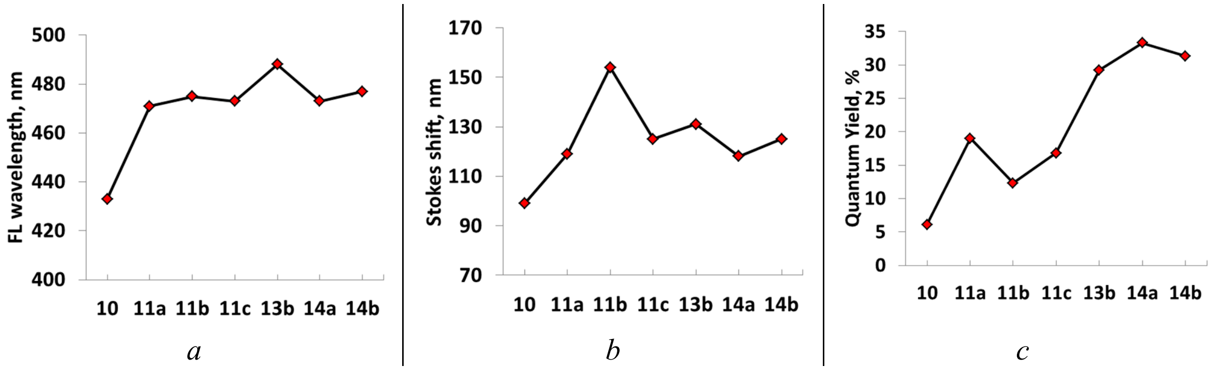

Figure S3. Influence of the structure onto the (*a*) maxima emission, (*b*) Stokes shift and (*c*) QYs DATs **10**, **11a**–**c**, **13b** and **14a**,**b**.

Table S3. Lifetime values of DATs **10**, **11a**–**c**, **13b** and **14a**,**b** obtained in $CDCl_3$.

Compd	τ_1[a] (ns)	A_1[b]	τ_2[a] (ns)	A_2[b]	$\langle\tau\rangle_f$[c] (ns)	χ^2[d]
**10**	1.09	0.78	3.46	0.21	1.577	1.06
**11b**	0.88	0.23	2.62	0.77	2.220	1.06
**11a**	0.80	0.23	0.24	0.77	0.369	1.08
**11c**	1.09	0.25	3.04	0.75	2.553	1.17
**14a**	1.33	0.46	3.14	0.54	2.307	1.13
**13a**	1.72	0.28	3.94	0.72	3.318	1.11
**14b**	1.50	0.39	3.60	0.61	2.781	1.05

[a] Fluorescence lifetime. [b] Fractional contribution. [c] Weighted mean lifetime. [d] χ^2-Chi-squared

Compound **10**

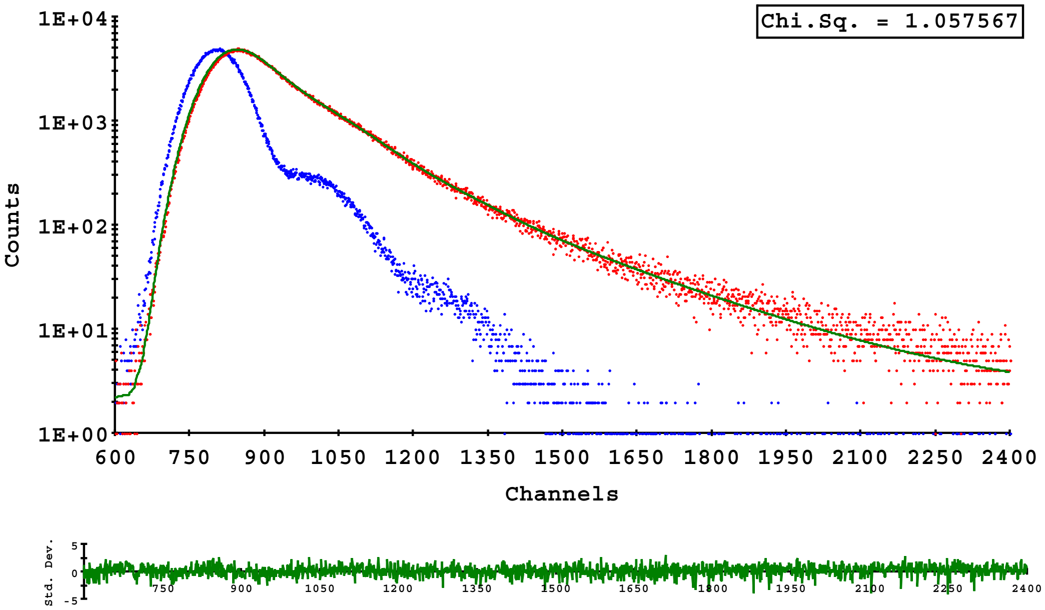

Compound **11a**

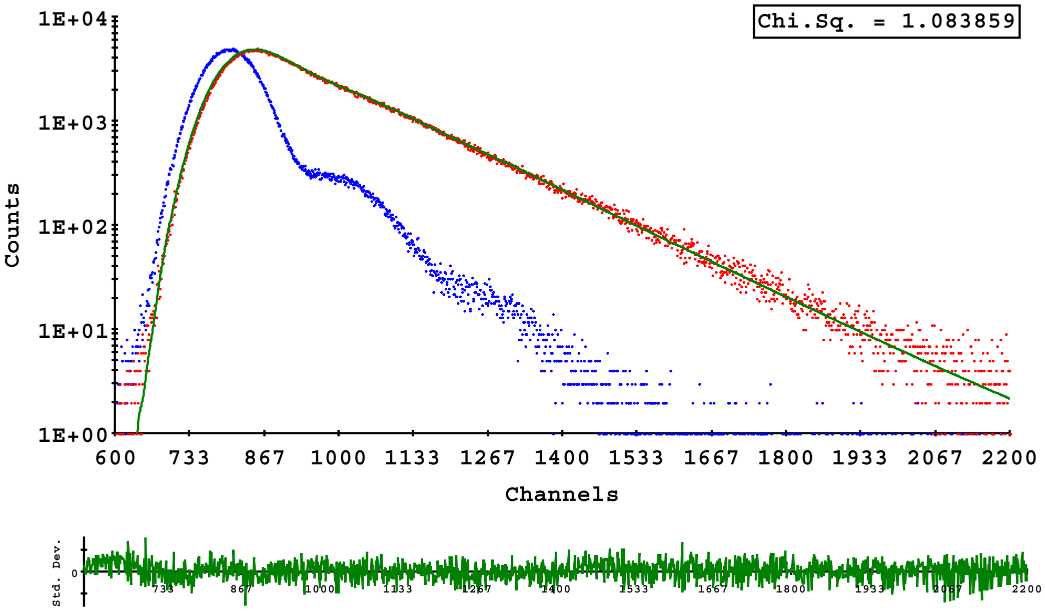

Compound **11b**

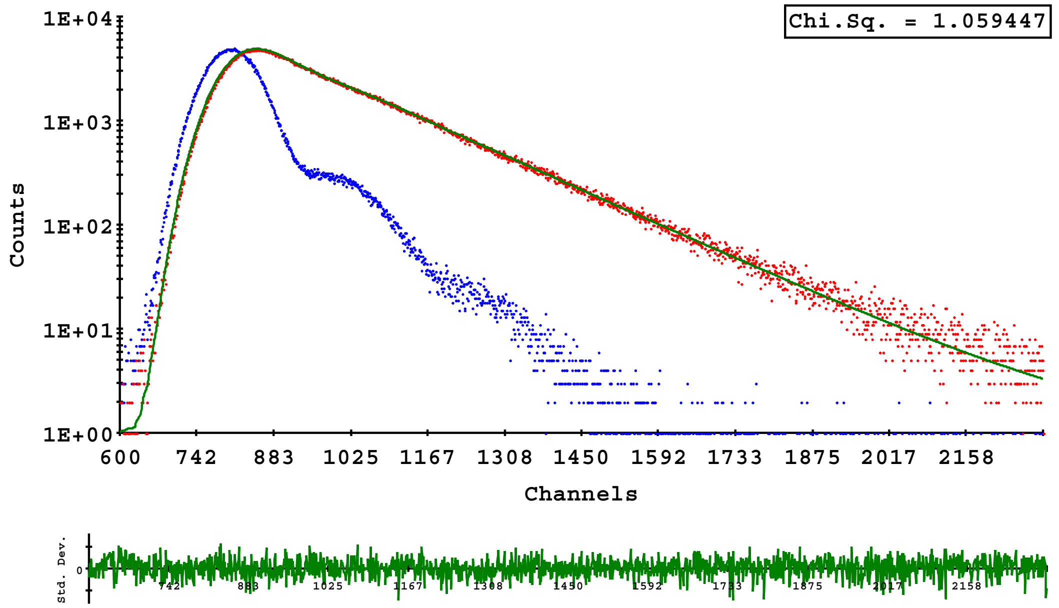

Compound **11c**

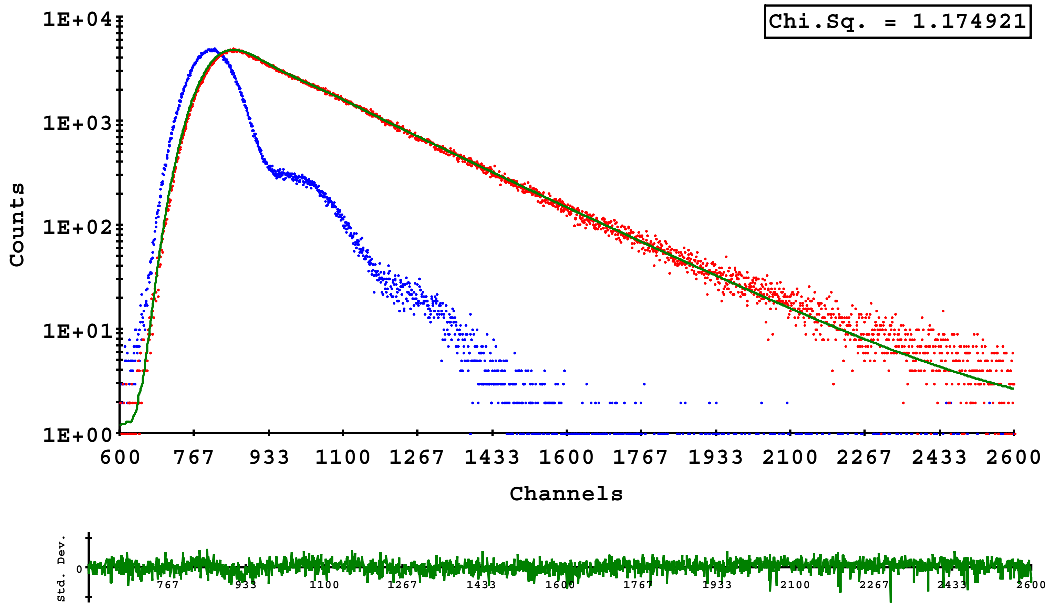

Compound **13a**

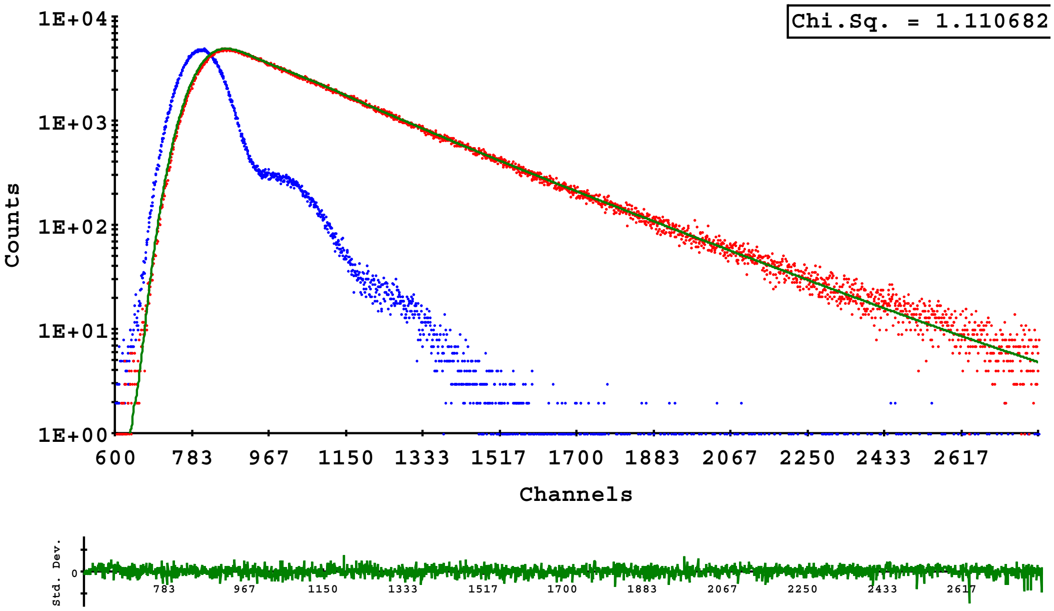

Compound **14a**

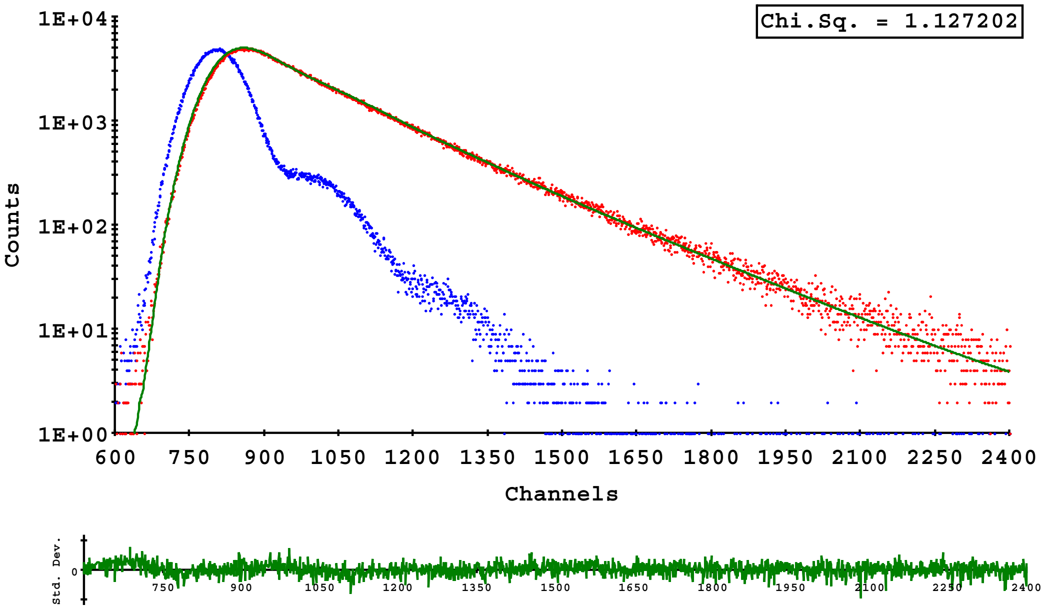

Compound **14b**

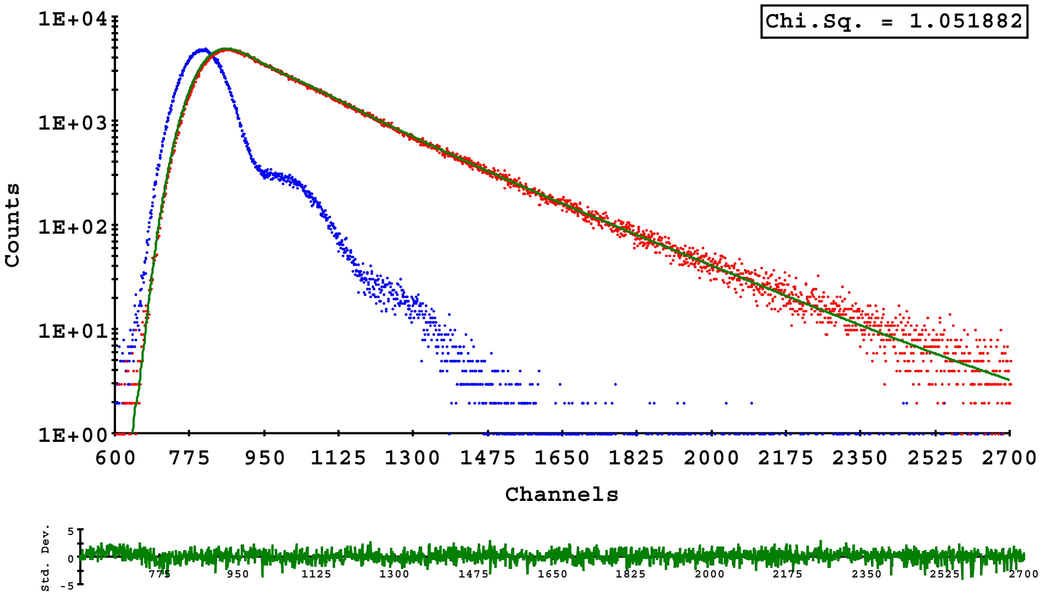

Figure S4. Fluorescence decay curves of DATs **10**, **11a–c**, **13a** and **14a**,**b** in $CDCl_3$.

Table S4. Photophysical data for DATs **10**, **11c**, **13a** and **14b** in solvents with different polarities

Entry	Compd.	Solvent	UV–Vis		Fluorescence		Stokes shift, nm/cm^{-1}
			λ_{max}, nm	ε, M^{-1}cm^{-1}	λ_{em}, nm	QY, %	
1	**10**	Toluene	334	19400	–	–	–
2		THF	339	23100	–	–	–
3		EtOH	340	20500	442	11	102/6787
4		MeOH	340	10780	447	8	107/7040
5		EG	341	10800	454	15	113/7299
6		$CHCl_3$	334	15200	433	6	99/6845
7		EtOAc	334	19200	–	–	–
8		DMF	343	17300	–	–	–
9		MeCN	335	18800	–	–	–
10		DMSO	344	10400	–	–	–
11		DMSO–H_2O	340	12300	459	9	119/7625
12	**11c**	Toluene	350	12600	470	27	120/7295
13		THF	353	15800	482	20	129/7582
14		EtOH	349	14200	491	4	142/8287
15		MeOH	349	12800	500	10	151/8653
16		EG	351	15800	491	4	140/8123
17		$CHCl_3$	348	15400	473	17	125/7594
18		EtOAc	349	14100	476	12	127/7645
19		DMF	352	15600	494	8	142/8166
20		MeCN	345	15500	495	5	150/8783
21		DMSO	356	15200	497	5	141/7969
22		DMSO–H_2O	352	14800	503	6	151/8528
23	**13a**	THF	358	14300	489	27	131/7483
24		EtOH	352	17200	495	9	143/8207
25		MeOH	350	15900	497	7	147/8451
26		EG	355	4900	500	9	145/8169
27		$CHCl_3$	357	13200	488	29	131/7519
28		EtOAc	353	14800	490	24	137/7920
29		DMF	358	16700	495	17	137/7731
30		MeCN	350	14500	496	20	146/8410
31		DMSO	361	16300	499	19	138/7661
32		DMSO–H_2O	355	18700	505	4	150/8367
33	**14b**	Toluene	355	14536	474	38	119/7072
34		THF	357	15713	488	28	131/7519
35		EtOH	352	16600	494	10	142/8166
36		EG	353	7400	498	17	145/8248
37		$CHCl_3$	352	17180	477	33	125/7445
38		EtOAc	353	17760	491	24	138/7962
39		DMF	358	16505	495	21	137/7731
40		MeCN	349	16737	492	17	143/8328
41		DMSO	362	17142	499	19	137/7584
42		DMSO–H_2O	358	18100	502	5	144/8013

Lippert-Mataga plot [4, 5]:

$$\tilde{\nu}_a - \tilde{\nu}_f = m_{L-M} f(\varepsilon, n) + constant \quad \text{(Eq. S1)}$$

m_{L-M} – the slope of the linear plot of the Stokes shift versus the orientation polarizability

$f(\varepsilon, n)$ – orientation polarizability of the solvents

$\tilde{\nu}_a - \tilde{\nu}_f$ – Stokes shift

$$f(\varepsilon, n) = \left(\frac{\varepsilon-1}{2\varepsilon+1} - \frac{n^2-1}{2n^2+1}\right) \quad \text{(Eq. S2)}$$

ε – dielectric constant, *n* – refractive index

Reichardt plot [6–8]:

$$\tilde{\nu}_a - \tilde{\nu}_f = m_{E_T^N} E_T^N + constant \quad \text{(Eq. S3)}$$

E_T^N – the normalized solvent polarity of Reichardt

$m_{E_T^N}$ – the slope of the linear plot of the Stokes shift versus the microscopic solvent polarity

$$E_T^N = \frac{E_T(30)solvent - E_T(30)TMS}{E_T(30)water - E_T(30)TMS} = \frac{E_T(30)solvent - 30.7}{32.4} \quad \text{(Eq. S4)}$$

$E_T(30)_i$ – the empirical solvent polarity parameter

TMS represent tetramethylsilane (non-polar solvent)

Dimroth-Reichardt plot [6–8]:

$$\tilde{\nu}_a - \tilde{\nu}_f = m_{E_{T(30)}} E_T\,(30) + constant \quad \text{(Eq. S5)}$$

$$E_T(30)_i = hcN\tilde{\nu}_{ai} = 2.8591x10^{-3}\tilde{\nu}_{ai}\ (in\ kcal\ mol^{-1}) \quad \text{(Eq. S6)}$$

$m_{E_{T(30)}}$ – the slope of the linear plot of the Stokes shift versus the empirical solvent polarity parameter

Table S5. Photophysical characteristic of DAT **10** in DMSO-water (c = 1 × 10^{-5} M) with varying amounts of water.

Entry	Amount of water, %	UV-Vis		Fluorescence		Stokes shift, nm/cm^{-1}
		λ_{max}, nm	ε, M^{-1}cm^{-1}	λ_{em}, nm	QY, %	
1	0	344	48400	–	–	–
2	10	340	55400	448	14	108/7090
3	20	340	45800	450	16	110/7190
4	30	340	44200	451	13	111/7239
5	40	340	43400	453	11	113/7337
6	50	340	42800	455	10	115/7434
7	60	340	42400	455	8	115/7434
8	70	340	45000	456	6	116/7482
9	80	340	43800	457	6	117/7530
10	90	340	41200	459	6	119/7625
11	99	340	45400	460/12	6	120/7673

Table S6. Photophysical characteristic of DAT **10** in THF-water (c = 1 × 10^{-5} M) with varying amounts of water.

Entry	Amount of water, %	UV-Vis		Fluorescence		Stokes shift, nm/cm^{-1}
		λ_{max}, nm	ε, M^{-1}cm^{-1}	λ_{em}, nm	QY, %	
1	0	335	31200	–	–	–
2	10	335	26800	441	10	106/7175
3	20	340	35400	442	13	102/6787
4	30	340	36800	443	12	103/6838
5	40	340	38000	444	11	104/6889
6	50	340	38000	445	10	105/6940
7	60	340	34400	446	9	106/6990
8	70	340	33200	449	9	109/7140
9	80	340	36400	454	8	114/7385
10	90	340	36800	456	7	116/7482
11	99	340	28000	456/15	7	116/7482

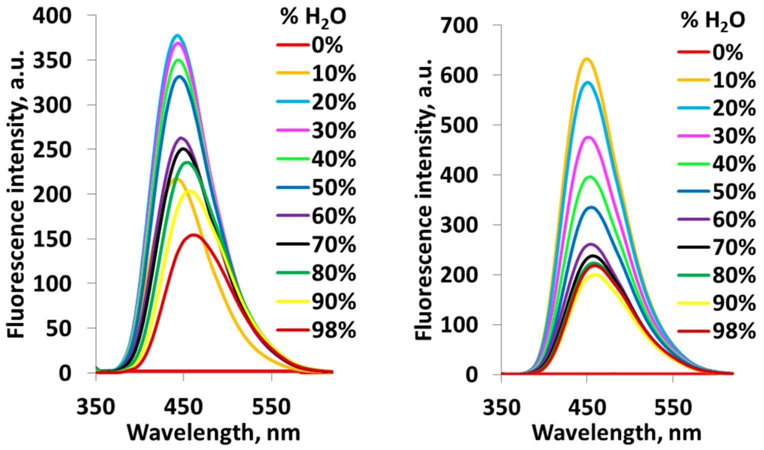

a *b*

Figure S5. Emission spectra DAT **10** in (*a*) DMSO and (*b*) THF with different portion of water.

Table S7. Photophysical characteristic of DAT **11c** in DMSO-water (c = 1 × 10^{-5} M) with varying amounts of water.

Entry	Amount of water, %	UV-Vis		Fluorescence		Stokes shift, nm/cm^{-1}
		λ_{max}, nm	ε, $M^{-1}cm^{-1}$	λ_{em}, nm	QY, %	
1	0	355	41000	496	4.9	141/8008
2	10	355	39200	497	10.2	142/8048
3	20	355	40600	499	10.3	144/8129
4	30	355	41800	500	9.6	145/8169
5	40	355	38600	501	9.3	146/8209
6	50	355	41400	503	7.4	148/8288
7	60	355	39000	505	6.4	150/8367
8	70	355	39000	505	5.6	150/8367
9	80	355	39000	505	5.1	150/8367
10	90	355	39200	505	4.6	150/8367
11	98	355	39200	505	3.8	150/8367

Table S8. Photophysical characteristic of DAT **11c** in THF-water (c = 1 × 10^{-5} M) with varying amounts of water.

Entry	Amount of water, %	UV-Vis		Fluorescence		Stokes shift, nm/cm^{-1}
		λ_{max}, nm	ε, $M^{-1}cm^{-1}$	λ_{em}, nm	QY, %	
1	0	352	34200	482	19.2	130/7662
2	10	352	32400	491	10.0	139/8042
3	20	352	32000	493	9.8	141/8125
4	30	352	33000	494	10.1	142/8166
5	40	352	33800	495	10.5	143/8207
6	50	352	34400	495	10.1	143/8207
7	60	352	32400	497	8.9	145/8288
8	70	352	34000	498	8.0	146/8329
9	80	352	35000	498	5.8	146/8329
10	90	352	35200	502	4.6	150/8489
11	99	352	32400	502	2.9	150/8489

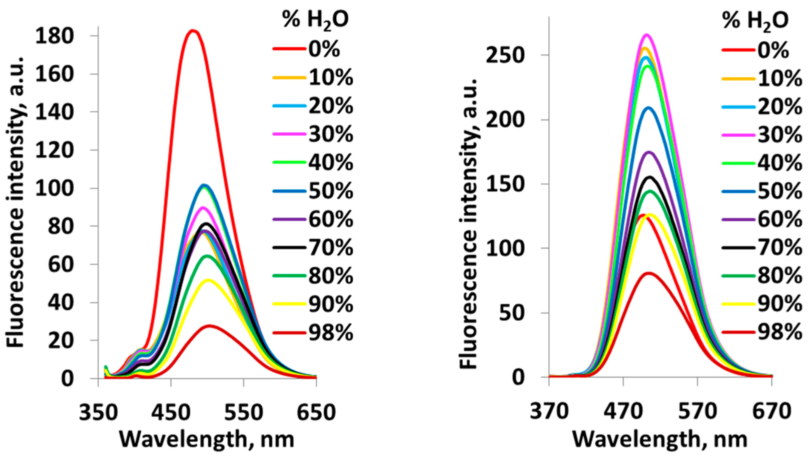

a *b*

Figure S6. Emission spectra DAT **11c** in (*a*) DMSO and (*b*) THF with different portion of water.

4. References

1. Dolomanov, O.V.; Bourhis, L.J.; Gildea, R.J.; Howard, J.A.K.; Puschmann, H. OLEX2: a complete structure solution, refinement and analysis program. J. Appl. Cryst., 2009, 42, 339–341.
2. Bourhis, L.J.; Dolomanov, O.V.; Gildea, R.J.; Howard, J.A.K.; Puschmann, H. The anatomy of a comprehensive constrained, restrained refinement program for the modern computing environment – Olex2 dissected. Acta Cryst. 2015, A71, 59–75.
3. Sheldrick, G.M. A short history of SHELX. Acta Cryst., 2008, A64, 112–122.
4. Sıdır, İ.; Sarı, T.; Sıdır, Y. G.; Berber H. Synthesis, solvatochromism and dipole moment in the ground and excited states of substitute phenol derivative fluorescent Schiff base compounds. J. Mol. Liquids, 2022, 346, 117075.
5. Lippert, E. Spektroskopische Bestimmung des Dipolmomentes aromatischer Verbindungen im ersten angeregten Singulettzustand. Z. Elektrochem., Ber. Bunsengesel. Phys. Chem., 1957, 61, 962–975.
6. Cerõn-Carrasco, J.P.; Jacquemin, D.; Laurence, C.; Planchat, A.; Reichardt, C.; Sraïdi, K. Solvent polarity scales: determination of new ET(30) values for 84 organic solvents. J. Phys. Org. Chem., 2014, 27, 512–518.
7. Reichardt, C. Empirical parameters of solvent polarity as linear free-energy relationships. Angew. Chem. Int. Ed., 1979, 18, 98–110.
8. Wang, Q.; Cai, L.; Gao, F.; Zhou, Q.; Zhan, F.; Wang, Q. Photochromism of Schiff base compounds derived from N,N'-bis(2-aminophenyl)isophthalamide: structure and photosensitivity. J. Mol. Struct., 2010, 977, 274–278.
